# On the Use of the Discrete Constant pH Molecular Dynamics to Describe the Conformational Space of Peptides

**DOI:** 10.3390/polym13010099

**Published:** 2020-12-29

**Authors:** Cristian Privat, Sergio Madurga, Francesc Mas, Jaime Rubio-Martínez

**Affiliations:** Department of Material Science and Physical Chemistry & Research Institute of Theoretical and Computational Chemistry (IQTCUB), University of Barcelona, C/Martí i Franquès 1, 08028 Barcelona, Spain; cprivat@ub.edu (C.P.); fmas@ub.edu (F.M.)

**Keywords:** constant pH molecular dynamics, AMBER, blocked tripeptides, Ramachandran maps

## Abstract

Solvent pH is an important property that defines the protonation state of the amino acids and, therefore, modulates the interactions and the conformational space of the biochemical systems. Generally, this thermodynamic variable is poorly considered in Molecular Dynamics (MD) simulations. Fortunately, this lack has been overcome by means of the Constant pH Molecular Dynamics (CPHMD) methods in the recent decades. Several studies have reported promising results from these approaches that include pH in simulations but focus on the prediction of the effective pKa of the amino acids. In this work, we want to shed some light on the CPHMD method and its implementation in the AMBER suitcase from a conformational point of view. To achieve this goal, we performed CPHMD and conventional MD (CMD) simulations of six protonatable amino acids in a blocked tripeptide structure to compare the conformational sampling and energy distributions of both methods. The results reveal strengths and weaknesses of the CPHMD method in the implementation of AMBER18 version. The change of the protonation state according to the chemical environment is presumably an improvement in the accuracy of the simulations. However, the simulations of the deprotonated forms are not consistent, which is related to an inaccurate assignment of the partial charges of the backbone atoms in the CPHMD residues. Therefore, we recommend the CPHMD methods of AMBER program but pointing out the need to compare structural properties with experimental data to bring reliability to the conformational sampling of the simulations.

## 1. Introduction

Nowadays, there are a large number of theoretical approaches that reproduce the behaviour of proteins in a multiple description level. In fact, these approaches along with experiments have unrevealed several mechanisms of action of biomolecules involved in physiological process and assisted in drug design projects for a therapeutic aim [1,2,3,4,5]. Among them, Molecular Dynamics (MD) is widely popular in the computational biochemistry field since it is capable of reproducing the interactions and conformations of a protein over time. Some studies have performed all-atom MD simulations that reaches the millisecond scale at the expense of a high computational cost [6]. To overcome the resource limitation, many efforts have been focused on the enhanced-sampling techniques, currently providing a wide range of them, such as accelerated MD [7], metadynamics [8,9], replica exchange MD [10], etc. Although these new methods have been successful for a better exploration of the conformational space of biochemical systems, it seems that other aspects to improve the accuracy of the simulations have not been as developed as the mentioned above, e.g., solvent pH, force field parameterization, or water models.

The conformational space of proteins is dependent on the solvent pH. Since the pH can change the protonation state of the amino acids and this configures the partial charges of the atoms, the solvent pH finally modulates the protein interactions and, therefore, its conformation. Moreover, it is well-known that physiological pH ranges from 4.5 in lysosomes up to 8.0 in the mitochondria [11], so it can notably vary depending on the cell’s organelle. In this context, the solvent pH may play a key role in the biological function of proteins. Its effect is especially important in proteins that have titratable amino acids in the active site or in those having pH-sensible domains, which can determine their activity or structure. However, the implementation of the solvent pH in simulations is far from trivial, but it seems necessary to understand in more detail the mechanism of action of the pH-sensible proteins. Conventional MD (CMD) simulations usually address this issue by only estimating the protonation state of the amino acids with pKa prediction models, such as PROPKA [12] or H++ [13], among other approaches [14], and fixing it during the simulation. Unfortunately, this assumption is not accurate enough for amino acids with an effective pKa close to the solvent pH given that CMD simulations only describe one of the protonation states over time. 

In the last decades, several efforts have been made to implement the pH effect in the simulations, finally developing the so-called Constant pH Molecular Dynamics (CPHMD) methods. There were many proposals that included the pH in simulations [15,16,17,18], but here, we highlight the two most successful methods in the present. On one hand, Brooks and co-workers [19] conceived the continuous CPHMD method [19,20,21,22,23] based on the λ-dynamics [24], in which the titration coordinate, λ, oscillates between 0 (protonated) and 1 (deprotonated state) in the potential function. On the other hand, methods based on stochastic processes to change the protonation state of the titratable residues appeared under the name of discrete CPHMD [25,26,27,28,29,30]. Baptista and co-workers [25] proposed the Monte Carlo (MC) and continuum electrics (CE) algorithm to simulate the protonation equilibrium, opening the door to a new branch of approaches. Both continuous and discrete CPHMD methods have shown promising results in predicting the pKa of amino acids in proteins or even in the conformational sampling [31,32,33,34,35,36]. However, deficiencies have also been reported, i.e., a lack of accuracy in the description of some physical properties or the trapping of the systems into a local minimum. Some reports pinpointed the strengths and weaknesses [37,38,39,40,41] of these methods as well as a few reviews compiled its development in the past [42,43]. Fortunately, CPHMD methods have improved by introducing modified force fields, water models, or other algorithm details, but adopting also enhanced-sampling approaches to get over the drawbacks [22,29,30,44,45,46,47,48,49]. 

These methods have been implemented in popular packages, such as CHARMM [50], GROMACS [51] or AMBER [52], but its usage is not extensive. The last package, which is extensively popular in the simulation of biomolecular systems, has available both the continuous and discrete CPHMD methods as well as the Replica Exchange pH Molecular Dynamics (REPH-MD) [30]. Given that the inclusion of the pH in simulations is increasingly common, this work wants to study the effect of the pH in biomolecular systems focusing on the accuracy of the discrete CPHMD method implemented in AMBER18 version [53] from a conformational perspective. To achieve this goal, microsecond simulations of blocked di(amino acids) tripeptides of six titratable amino acids have been performed using a Generalized Born model for implicit solvation in both CMD and CPHMD methods. Depending on the method, the systems have been built in such a way that they are equivalent to each other. The protonatable systems have been performed in strong acid or basic pH conditions to ensure a full protonated or deprotonated state, whereas conventional simulations have been carried out with a fixed protonation state. Thus, the Ramachandran plots and the energy contributions of small peptides are analyzed using both conventional and constant pH Molecular Dynamics calculations. The results of the simulations are discussed in this work, revealing some merits and weaknesses of the CPHMD implementation in the AMBER18 version.

## 2. Materials and Methods

**Figure 1:**
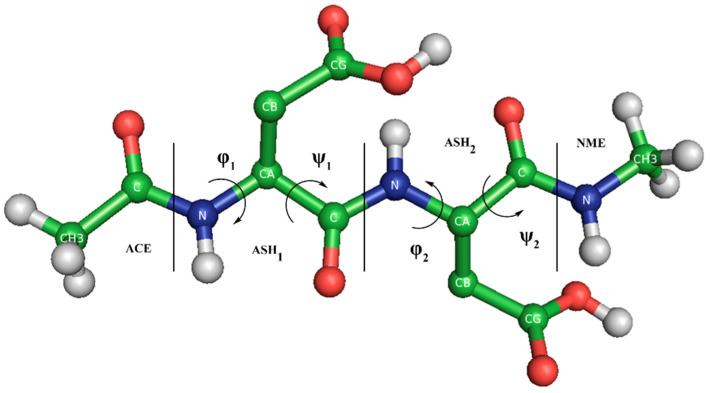
Blocked Asp_2_ tripeptide in the syn-O2 protonated state. Amino acids, capping groups, and φ and ψ dihedral angles are indicated. The θ angle is constructed by the CG_1_, CA_1_, CA_2_, and CG_2_ atoms. Nonpolar hydrogens of the amino acids are hidden. Subscripts refer to monomer 1 and 2.

**Blocked Tripeptide Building.** Tripeptides (ACE-X-X-NME, hereinafter X_2_) capped on the extremes by the acetyl (ACE) and N-methyl (NME) groups were built for the protonated, deprotonated and titratable forms of X, with X = {lysine, tyrosine, cysteine, histidine, glutamic, aspartic} amino acids (Figure 1). The LYN, CYM, HID, HIE, GLU, and ASP residues were used for the deprotonated forms while LYS, TYR, CYS, HIP, GLH and ASH for the protonated ones in the CMD simulations. The titratable (or CPHMD) systems were built using the LYS, TYR, CYS, HIP, GL4 and AS4 residues (using the AMBER convention). ff14SB force field [54] and constph.lib (in CPHMD) were loaded in the LEaP module of AMBER18. Next, the CPIN file was generated for the titratable systems, specifying the initial protonation state according to the solvent pH and the Generalized Born (GB) model of Onufriev et al. [55] (igb = 2). The lysine, tyrosine, and cysteine amino acids have two possible protonation states: the de- and protonated forms. The histidine possesses up to three protonation states classified in the de- and protonated form: the doubly protonated HIP state for the protonated form and the ε- (HIE) and δ- (HID) states for the deprotonated histidine. HIE and HID are defined by the position of the remaining hydrogen, at the N-epsilon or N-delta nitrogen, respectively, after deprotonation. The δ-state was chosen as the initial protonation state for the CPHMD simulations of histidine. Finally, the glutamic and aspartic acids can be found in the deprotonated form or up to four states in the protonated form. These protonated states depend on the position (syn- or anti-) when one of the two oxygens (O1 or O2) of the carboxyl group is protonated. Figure S1 illustrates the four protonatable sites of the side chain of the AS4 residue. State 1 (syn-O2 protonation) was chosen for the initial protonated state in the CPHMD simulations, which is the default protonated state in the CMD method. Counterions were considered implicitly with an ionic strength of 0.1 M in the solvation model.

**Simulations**. Each system was minimized following a three-stage protocol with different restraints: (i) on all atoms, (ii) only on the backbone atoms, and (iii) on the free system. 5000 steps (maximum) of steepest descent method per stage were performed. Restraints were introduced with force constants of 5.0 kcal/mol·A^−2^. In the titratable systems, the implicit CPHMD method (icnstph = 1) was turned on to define the protonation state of the amino acids but without any change on the protonation states (ntcnstph > 5.000).

After the minimization step, a heating simulation was performed by linearly increasing the temperature (1 K·ps^−1^) of the blocked tripeptide up to 300 K. Then, the system was equilibrated by keeping the tripeptide for 200 ps at 300 K. To increase the conformational sampling [56], four replicates were generated for each system, using the final coordinates of the equilibration step but resetting the initial velocities. Production runs of 1 µs per replicate were performed with the implicit solvent method, using the Generalized Born (igb = 2) model and an ionic strength of 0.1 M. The SHAKE algorithm constrained the bond lengths. Langevin thermostat with a collision frequency of 3 ps^−1^ was selected for the thermal bath and no periodic boundary conditions (PBC) were required. For the titratable simulations, an implicit CPHMD method was used with a trial protonation state change frequency of 0.01 ps^−1^ (ntcnstph = 5). Strong pH conditions were set to obtain a dominant protonation state during CPHMD simulations. pH values of 12.0 and 1.0 for de- and protonated forms were chosen, respectively. The only exception was the blocked Lys_2_ tripeptide, which required a higher basicity in the solvent (pH 14.0). Table 1 collects the residue type, method and pH of the simulations.

**Energetic and Conformational Analysis.** Energies, coordinates, and output files were updated every 2, 10, and 20 ps, respectively. Energy terms and normalized histograms of each term were calculated with the CPPTRAJ module [57]. Dihedral angles (φ, ψ and an angle related to the orientation of the side chains with respect to the Cα atoms, which is called the “θ angle” from now) were also extracted with CPPTRAJ. An in-house tool transformed the dihedral angles generated during the simulation into Gibbs free energies with Equation (1) allowing building the Ramachandran potential energy surface.

(1) $$∆G=-K_{B}Tln\left(n_{i}/n_{max}\right)$$

where *k_B_* is the Boltzmann constant, *T* is the temperature, and *n_max_* and *n_i_* are the maximum population and the population of a cell *i* in a grid of the dihedral angles with a spacing of 1°. The Ramachandran map was separated into nine conformational regions (C_5_, P^II^, αD, β2, C7axial, αL, α’, αR and C7eq) according to Rubio-Martinez et al. [58] (Figure S2), and the global populations in each conformational region were calculated. Each amino acid was analyzed separately, leading into two sets of results that correspond to the N-terminal amino acid (set 1) and the C-terminal amino acid (set 2). Minima were also located but using a wider grid spacing (2°) to decrease the apparition of false minima. All plots were generated with GNUPLOT (version 4.6) [59].

## 3. Results

### 3.1. Gibbs Free Energies in the Ramachandran Space

The conformational sampling of each system was analyzed by means of the Ramachandran map. Since the blocked tripeptides have two amino acids with their φ and ψ backbone dihedral angles (Figure 1), the representation of the pair φ/ψ angles of each monomer (the N-terminal and C-terminal amino acid) was done. The description of the results of the MD simulations starts with the amino acids that have basic pKa values, continues by the specific case of the histidine and, finally, ends with those with a carboxyl group in the side chain.

#### 3.1.1. Basic pKa Amino Acids

**Figure 2:**
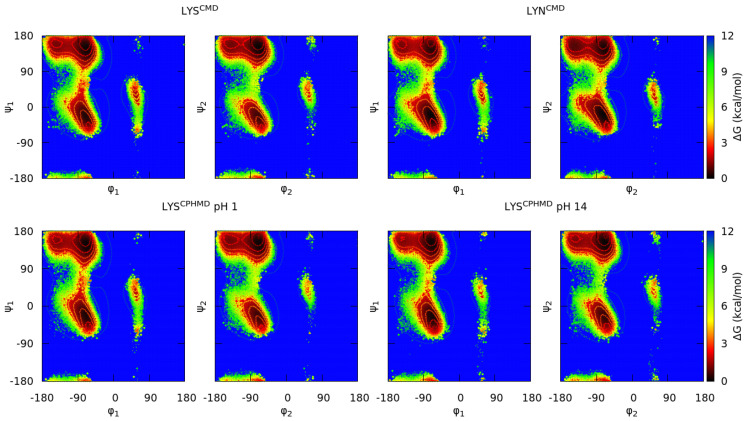
Gibbs free energies in the Ramachandran space of the blocked Lys_2_ tripeptide. Each subtitle indicates the residue, the simulation method (in the superscript), and the pH (only for the CPHMD simulations). Both sets of dihedrals (φ_1_/ψ_1_ from the N-terminal amino acid; φ_2_/ψ_2_ from the C-terminal amino acid) are illustrated. Protonated forms are in the left (CMD; top—CPHMD; bottom) and deprotonated ones in the right (CMD; top—CPHMD; bottom). Solid lines indicate an increase of 0.6 kcal/mol of the energy values.

We include in this group those protonatable amino acids with an intrinsic pKa greater than 7.0. The conformational sampling of this set of blocked tripeptides is represented in the Ramachandran maps for each simulation conditions (CMD at the top and CPHMD at the bottom of each figure). The LYS systems are illustrated in Figure 2, and TYR and CYS are found in the Supplementary Materials (Figures S3 and S4). The deprotonated form of tyrosine is not available in the AMBER libraries for CMD method; thus, only the simulations of the protonated form were performed. However, charges of deprotonated tyrosine can be calculated, since it has been proven that it can play an important role in the conformation of some proteins [60].

**Figure 3:**
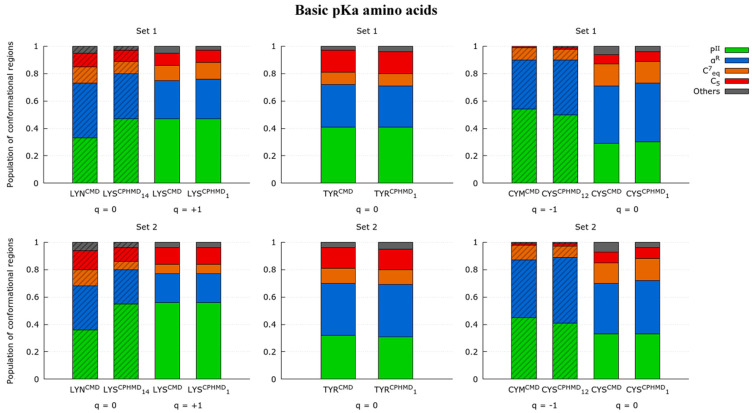
Ratio of the fourth most populated conformational regions (P^II^, α^R^, C^7^_eq_, and C_5_ in green, blue, orange, and red, respectively) in the Ramachandran map of the LYS, TYR, and CYS amino acids. The labeling indicates the residue, the simulation method (in the superscript), and the pH (in the subscript, only for the CPHMD simulations). Subtitles indicate the set of dihedrals corresponding to monomer 1 (N-terminal) or monomer 2 (C-terminal amino acid). The total charge of the tripeptide is indicated below the systems (q = −1, 0 or +1). The box style (striped or solid) indicates those systems in the same protonation state, independently of the method used. The ‘others’ classification (gray) includes the conformational regions β_2_, α’, α_D_, α_L_, and C_7_^axial^.

The comparison of both simulation methods in the Ramachandran plots shows that LYS protonated forms (LYS^CMD^ and LYS^CPHMD^ at pH 1) are in agreement. Instead, the deprotonated simulations (LYN^CMD^ and LYS^CPHMD^ at pH 14) present smooth differences in the depth of the minima. For a further comprehension, the conformational profile of the blocked tripeptides was studied by delimitating the Ramachandran map in nine regions according to Rubio-Martinez et al. [58], which are related with a certain conformation (C_5_, P_II_, α_D_, β_2_, C_7_^axial^, α_L_, α’, α_R_, and C_7_^eq^). By computing the population of each region, the conformational propensities of each amino acid are estimated. The population ratios allow quantitatively comparing the conformational sampling of the simulation methods by identifying the most stable regions. The populations of these regions for each monomer are illustrated in Figure 3. In general, the P^II^ and α^R^ conformations prevail among all the others. For the LYS system, it is seen that protonated form shows close population ratios between their counterparts (LYS^CMD^ and LYS^CPHMD^ at pH 1). The deprotonated CMD simulations (LYN^CMD^) have a different population profile with respect to the other systems, showing a behavior far from the CPHMD counterpart (LYS^CPHMD^ at pH 14). In contrast, LYS^CPHMD^ at pH 14 has similar conformational populations with respect to LYS^CMD^ and LYS^CPHMD^ at pH 1.

A good correspondence for the protonated systems of the TYR amino acid (TYR^CMD^ and TYR^CPHMD^ at pH 1) is observed (Figure S3). Except for barely appreciable differences in the populations of minor conformational regions (C_7_^axial^ and α_L_), the Ramachandran maps and the population ratios (Figure 3) are in great agreement. Regarding the CYS systems (Figure S4), the conformational profiles show a similar trend to the TYR systems. Therefore, a good agreement between CMD and CPHMD counterparts is also observed in the Ramachandran maps (Figure S4) and population ratios (Figure 3) for the protonated (CYS^CMD^ and CYS^CPHMD^ at pH 1) and deprotonated forms (CYM^CMD^ and CYS^CPHMD^ at pH 12).

These observations proved that the CPHMD method was generally consistent in the conformational sampling of these amino acids, except for the deprotonated LYS form. Thus, a first weakness is identified given that Ramachandran maps of the deprotonated LYS^CPHMD^ system were unable to reproduce the conformational profile of the well-established CMD method.

#### 3.1.2. Histidine

This amino acid presents pKa values of 6.5 and 7.1 according to the protonation state. Depending on the position of the remaining hydrogen in the deprotonated form, the histidine can reach the δ- (N-delta atom) or the ε- (N-epsilon atom) state. Thus, two protonation states coexist when the imidazole ring of the side chain becomes neutral, modulating the conformational sampling of the system depending on the position of the hydrogen during the simulation.

**Figure 4:**
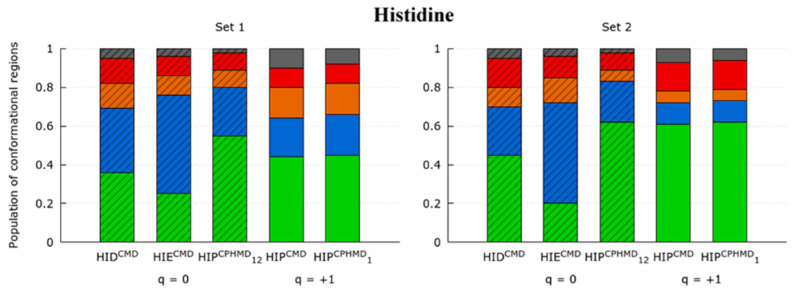
Ratio of the fourth most populated conformational regions (P^II^, α^R^, C^7^_eq_, and C_5_ in green, blue, orange, and red, respectively) in the Ramachandran map of the HIS amino acid. Each system indicates the residue, the simulation method (in the superscript), and the pH (in the subscript, only the CPHMD simulations). Subtitles are the set of dihedrals for the monomer 1 (N-terminal) or 2 (C-terminal amino acid). Total charge of the tripeptide is indicated below the systems (q = −1, 0 or +1). The box style (striped or solid) indicates those systems in the same protonation state, independently of the method used. The ‘others’ classification (gray) includes the conformational regions β_2_, α’, α_D_, α_L_ and C_7_^axial^.

The Ramachandran maps of HIS systems (Figure S5) illustrate the conformational sampling obtained from the simulations. The protonated systems (HIP^CMD^ and HIP^CPHMD^ at pH 1) show similar conformational profiles in the Ramachandran maps. The population ratios corroborate this observation: the HIP^CMD^ and HIP^CPHMD^ at pH 1 systems present close population ratios in Figure 4. In contrast, the deprotonated simulations (HIE^CMD^, HID^CMD^ and HIP^CPHMD^ at pH 12) show differences in the depth of minima of the Ramachandran plots. Moreover, the population ratios of the CMD simulations (HIE^CMD^ and HID^CMD^) are not in agreement with the HIP^CPHMD^ at pH 12 system. In this case, the HIP^CPHMD^ simulation at basic conditions presents population ratios closer to the protonated form rather than its CMD counterpart. The population ratios of the HIE^CMD^ and HID^CMD^ are far from being similar, which suggest that the position of the remaining hydrogen in the N-epsilon and N-delta atom plays an important role on the conformational sampling of the deprotonated forms.

While protonated forms are in good agreement from a conformational point of view, the deprotonated forms of histidine evidence that the CPHMD method in basic conditions is unable to reproduce the conformational sampling of the CMD counterparts. Since HIP^CPHMD^ at pH 12 coexists between the delta and epsilon protonation state in the neutral form, it could be expected as a population profile as a result of the combination of the profiles of both states. Instead, the P^II^ conformation of the CPHMD systems in basic conditions behaves similarly to the protonated simulations, which is a fact that is also observed in the LYS systems.

#### 3.1.3. Acidic Amino Acids

The glutamic and aspartic acids are two amino acids characterized by the four protonatable sites in the carboxyl group. Although both residues are similar, except for the pKa shift due to the additional methyl group in the GLU side chain, the Ramachandran maps and the population ratios do not behave similarly.

**Figure 5:**
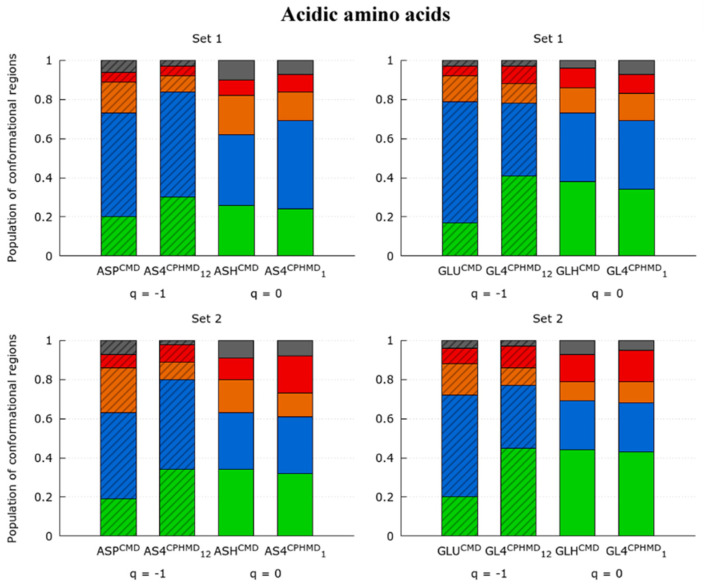
Ratio of the fourth most populated conformational regions (P^II^, α^R^, C^7^_eq_, and C_5_ in green, blue, orange, and red, respectively) in the Ramachandran map of the ASP and GLU amino acids. The labeling indicates the residue, the simulation method (in the superscript), and the pH (in the subscript, only for the CPHMD simulations). Subtitles indicate the set of dihedrals corresponding to monomer 1 (N-terminal) or monomer 2 (C-terminal amino acid). Total charge of the tripeptide is indicated below the systems (q = −1, 0 or +1). The box style (striped or solid) indicates those systems in the same protonation state, independently of the method used. The “others” classification (gray) includes the conformational regions β_2_, α’, α_D_, α_L_, and C_7_^axial^.

On one hand, the conformational samplings of the GLU systems illustrated in the Ramachandran maps (Figure S6) keep the trend of the results observed in the LYS and HIS systems. The Ramachandran plots and population ratios of the protonated simulations (GLH^CMD^ and GL4^CPHMD^ at pH 1) are in a good correspondence (Figure S6 and Figure 5). In contrast, this is not observed for the deprotonated systems (GLU^CMD^ and GL4^CPHMD^ at pH 12), whose population ratios significantly differ from each other. In fact, it is shown that GL4^CPHMD^ at pH 12 presents a similar population profile with respect to the GLH^CMD^ and GL4^CPHMD^ at pH 1. This fact is no longer surprising, since it also occurs in previous systems (LYS and HIP).

**Figure 6:**
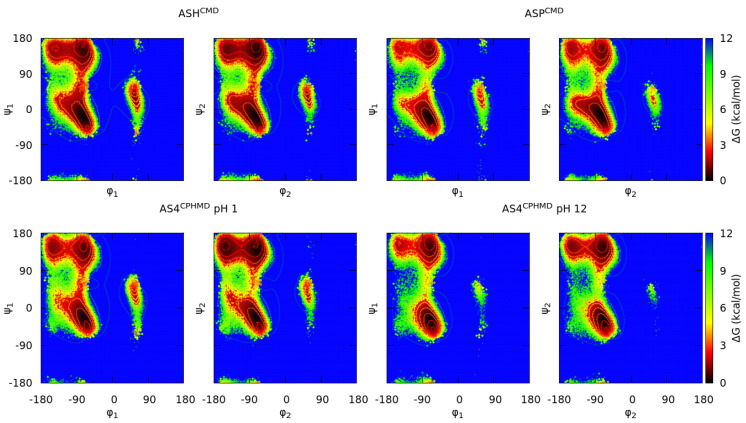
Gibbs free energies in the Ramachandran space of the blocked Lys_2_ tripeptide. Each subtitle indicates the residue, the simulation method (in the superscript), and the pH (only for the CPHMD simulations). Both sets of dihedrals (φ_1_/ψ_1_ from the N-terminal amino acid; φ_2_/ψ_2_ from the C-terminal amino acid) are illustrated. Protonated forms are on the left (CMD; top—CPHMD; bottom) and deprotonated ones are on the right (CMD; top—CPHMD; bottom). Solid lines indicate an increase of 0.6 kcal/mol of the energy values.

On the other hand, the ASP systems stand out given that protonated simulations (ASH^CMD^ and AS4^CPHMD^ at pH 1) show a mild disagreement in the minima of the Ramachandran maps (Figure 6) but not as significant as the deprotonated ones. However, the population ratios (Figure 5) confirm that this disagreement is due to smooth differences in the population of each conformation (including C_7_^eq^ and C_5_). The deprotonated systems of the ASP amino acid (ASP^CMD^ and AS4^CPHMD^ at pH 12) show a larger dissimilarity in the Ramachandran maps and population ratios.

Apart from the differences in the deprotonated forms, which are also observed in the previous sets, another element apparently interferes by causing small changes in the conformational sampling of protonated forms. These differences could be associated with the multiple protonatable position of the hydrogen when the amino acids are protonated. Moreover, the conformational sampling of ASP is probably more sensible to the position of the proton given that the carboxyl groups of the two consecutive ASP amino acids are closer between them if it is compared to the GLU systems, which have an additional methyl group in the side chain.

The Ramachandran plots proved the consistency of the CPHMD method when reproducing the conformational sampling of the protonated forms of the basic pKa, histidine, and acidic amino acids. However, some inconsistencies were identified for the deprotonated forms of all systems (except for CYS).

The main reason for this inconsistency in the deprotonated forms is the mismatch in the partial charges when comparing the CMD and CPHMD counterparts. Table S1 collects the partial charges of each amino acid atoms. In fact, AMBER manifested that CPHMD residues always use the partial charges of the protonated form, named a reference residue, in the backbone atoms and only changes the charges of the side chain atoms when the residue reaches another protonation state [26]. Thus, it is understandable that the electrostatics interactions are not totally reproducible using the CPHMD method. We hope this limitation can be solved in future updates of the method.

On the other hand, the attachment of the hydrogens in the CMD simulations with respect to the protons in the CPHMD method is another reason of the observed discrepancies. The residues of the CPHMD method always possess a hydrogen atom in all the protonatable sites during the simulation and activate them (by changing the partial charges of the side chain) according to the protonation state. Under this consideration, there are two scenarios: (i) the histidine and (ii) the acidic amino acids. For the histidine, the protonated form of the CPHMD method has the two hydrogens activated as the reference residue (HIP) of the CMD method, so there is no difference between them. Thus, the conformational samplings of the protonated simulations are in great agreement. However, the deprotonated forms of HIS have different protonation state samplings. The HIP^CPHMD^ at pH 12 simulations coexist in the δ- and ε-state over time, while the CMD method only establishes one of the two states (HIE or HID) in the simulation. Apart from the failure on reproducing the electrostatics due to the partial charges, the deprotonated forms of histidine are not entirely comparable because of the change of position of the activated hydrogen during the CPHMD simulations. The change of position of the hydrogen atom in the CPHMD simulations leads to different conformational sampling with respect to the CMD simulations, in which the hydrogen is fixed at the N-delta or N-epsilon atom positions.

The acidic amino acids present a similar problem but in the protonated forms. These residues have four protonatable sites (the anti- or syn- position in each oxygen of the carboxyl group), supposing a major allocation of the hydrogen when is protonated in comparison with the CMD system, in which the hydrogen is attached in the syn-O2 position. In fact, the populations of the protonation states during the CPHMD simulation were 96% and 4% (in average) for the syn- and anti- position, respectively, but counting that the hydrogen can be found in one of the two oxygens. In the CMD simulations, the hydrogen is bound to the O2 oxygen atom. In the CMD simulations, the change of position of the hydrogen is reached by the rotation of the bonds of the carboxyl group, which is more expensive if it is compared to the CPHMD method. Then, the CPHMD simulations at pH 1 are not totally comparable to the protonated CMD simulations due to the different sampling of the protonation states in both methods. However, the multiple protonatable positions of the CPHMD simulations is far from causing significant deviations in the conformational sampling of the acidic amino acids, as it can be observed in the Ramachandran maps and population ratios reported above.

### 3.2. Energetic Contributions

The energy terms of the AMBER’s force field form will provide more information to understand the divergence in the conformational sampling. Thus, normalized distributions of energies (total, kinetic, and potential, and each term of the potential energy) were computed using the energy values from the simulation and plotted with GNUPLOT [59]. Furthermore, the division of the electrostatic energy into backbone and side chain contributions were also performed with CPPTRAJ [57], allowing clarifying the implications of the mismatch of partial charges. This section is mainly focused on the electrostatics contribution, but other energy terms are also illustrated, and a few internal energies are highlighted during the explanation.

**Figure 7:**
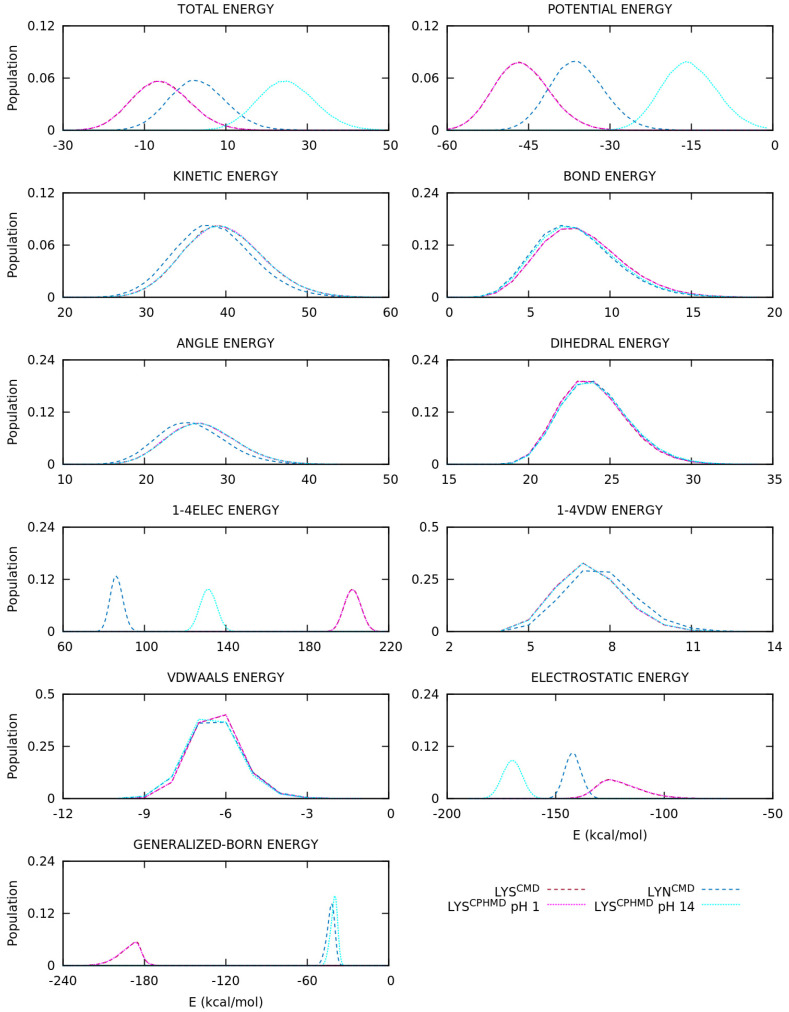
Energy distributions of the blocked Lys_2_ tripeptide. Global, inner, van der Waals, and electrostatics terms are illustrated. Dotted and dashed lines are CPHMD and CMD simulation methods, respectively.

**Figure 8:**
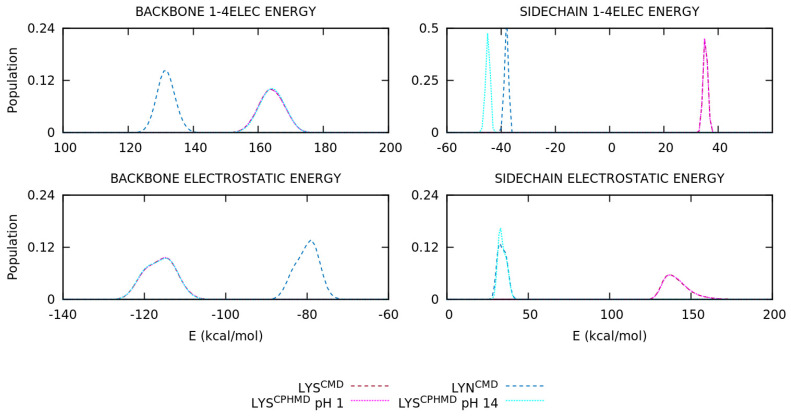
Energy distribution of the 1–4 and long-range electrostatics of the backbone and side chain atoms of the blocked Lys_2_ tripeptide. Dotted and dashed lines are CPHMD and CMD simulation methods, respectively.

The energy distributions of the basic pKa amino acids are illustrated in Figure 7 and Figures S7 and S8 for the LYS, TYR, and CYS systems, respectively. For the LYS system, the overlapping of protonated simulations (LYS^CMD^ and LYS^CPHMD^ at pH 1) is observed in all energy terms of Figure 7. On the contrary, the deprotonated LYS systems (LYN^CMD^ and LYS^CPHMD^ at pH 14) show a significant shift in the 1–4 electrostatic interactions, as well as the long-range electrostatics, which also present a different shape in the distribution. To understand the effect of the partial charges restriction in the implementation of the CPHMD method, the electrostatics terms of all the systems were decomposed into backbone and side chain atoms. The separation of the electrostatics in the LYS systems (Figure 8) illustrates that the contribution of the protonated systems (LYS^CMD^ and LYS^CPHMD^ at pH 1) perfectly overlaps in all parts of the amino acid. However, a discrepancy in both electrostatic terms of the backbone and the 1–4 electrostatics of the side chain distributions of the deprotonated simulations (LYN^CMD^ and LYS^CPHMD^ at pH 14) is observed. The deviation in the side chain electrostatics is possibly related to the partial charge of the C_β_ atom (Table S2).

For the TYR system, only the energy distributions of the protonated simulations (TYR^CMD^ and TYR^CPHMD^ at pH 1) are available in Figure S7. Both distributions perfectly overlap, as well as the electrostatics decomposition in the Figure S9. These results are in line with those ones observed in the Ramachandran maps. On the other hand, the energy distributions of the CYS systems (Figure S8) also present a good overlapping in the protonated simulations (CYS^CMD^ and CYS^CPHMD^ at pH 1). However, the deprotonated systems (CYM^CMD^ and CYS^CPHMD^ at pH 12) show mild shifts in the total, potential, dihedral and 1–4 electrostatics energies, and different shapes in the 1–4 and long-range electrostatics. The decomposition of electrostatics (Figure S10) in the deprotonated simulations evidences a modest shift in the distributions of the electrostatics in both side chain and backbone atoms. The backbone electrostatics of CYS^CPHMD^ at pH 12 suggests that the deprotonated form modulates the conformational sampling in such a way that the distribution shape finally becomes similar to CYM^CMD^. Furthermore, the conformational samplings of the deprotonated CYS systems (CYM^CMD^ and CYS^CPHMD^ at pH 12) (Figure S4 and Figure 3) are surprisingly in agreement, although some energy terms are not.

The protonated simulations of the HIS amino acid (HIP^CMD^ and HIP^CPHMD^ at pH 1) show a large overlap of the energy distributions in Figure S11. However, the deprotonated forms (HIE^CMD^, HID^CMD^ and HIP^CPHMD^ at pH 12) present dissimilarities in several energy terms (i.e., total energy, potential energy, electrostatics, and inner energies). The distribution of the CPHMD systems does not reproduce the δ- or ε- state of the neutral HIS, as it is seen in the plots. This observation was expected given that CPHMD simulations coexist between the two protonation states. Instead, the electrostatics of HIP^CPHMD^ at pH 12 show two peaks, representing these states, but outside the energy range of the deprotonated CMD forms. To unravel this behavior, the decomposition of electrostatics is illustrated in Figure S12. The distributions of the protonated simulations (HIP^CMD^ and HIP^CPHMD^ at pH 1) are in line with the observed trend of the global electrostatics. On the contrary, the deprotonated simulations (HIE^CMD^, HID^CMD^ and HIP^CPHMD^ at pH 12) show different distributions in all the contributions. The backbone electrostatics show that distributions of HIP^CMD^ and HIP^CPHMD^ at pH 1 and 12 overlap between them while the HIE^CMD^ and HID^CMD^ systems present their singular distribution. The side chain contributions are more coherent given that the distribution is closer to the deprotonated simulations (HIE^CMD^ and HID^CMD^) rather than the protonated ones (HIP^CMD^ and HIP^CPHMD^ at pH 1). Focusing on the deprotonated CPHMD system, the observed behavior in the backbone atoms is explained by the incorrect assignment of partial charges. The discrepancy of side chain atoms electrostatics is due to a sum of two factors: (i) the charge of the side chain atoms varies over time due to the CPHMD simulation reaching one of both delta and epsilon neutral states during the simulation, modulating the conformational sampling at the same time, and (ii) the distributions of the electrostatics decompositions for the HIP^CPHMD^ at pH 12 are calculated using the fixed partial charges of the HID or the HIE state in the CPHMD method without taking into account the real protonation state of the CPHMD system during the simulation. Then, these distributions of the HIP^CPHMD^ at pH 12 systems are approximated.

**Figure 9:**
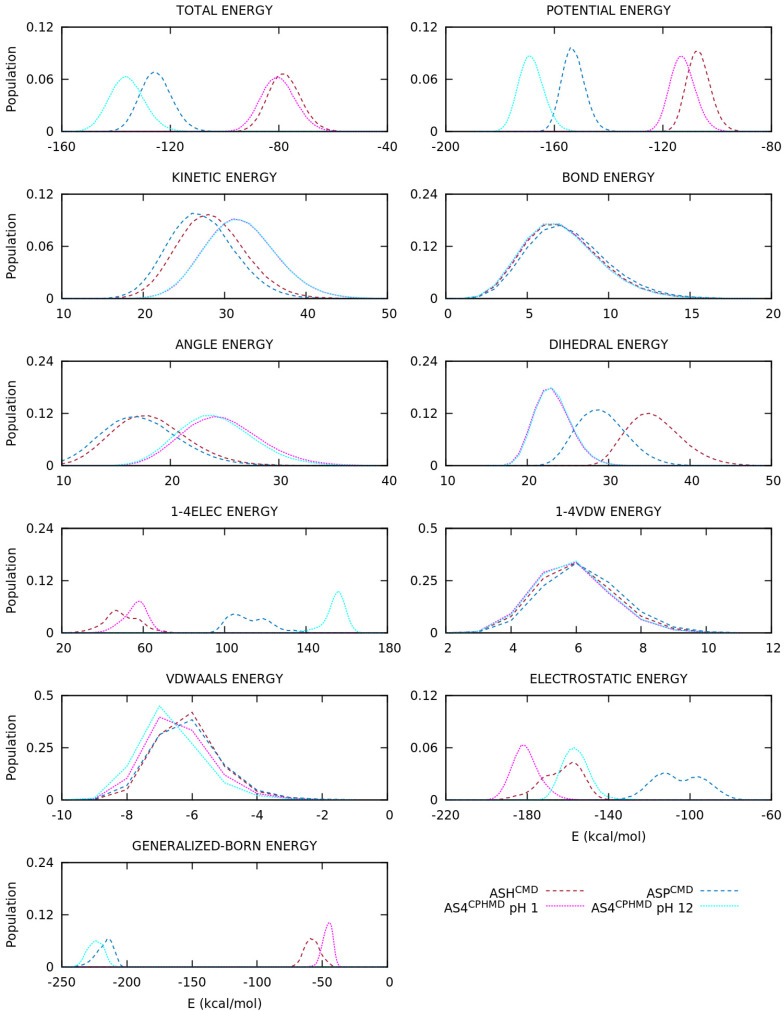
Energy distributions of the blocked Asp_2_ tripeptide. Global, inner, van der Waals, and electrostatics terms are illustrated. Dotted and dashed lines are CPHMD and CMD simulation methods, respectively.

**Figure 10:**
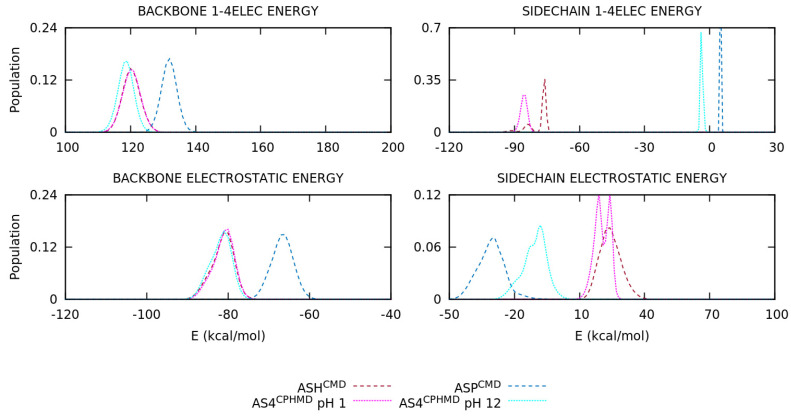
Energy distribution of the 1–4 and long-range electrostatics of the backbone and side chain atoms of the blocked Asp_2_ tripeptide. Dotted and dashed lines are CPHMD and CMD simulation methods, respectively.

The ASP and GLU amino acids introduce the multiple protonatable sites in the CPHMD simulations. The energy distributions are illustrated in Figure 9 and Figure S13, respectively. For the ASP amino acid, the energy distributions of the protonated systems (ASH^CMD^ and AS4^CPHMD^ at pH 1) do not overlap, unlike the previous sets, due to the electrostatics (1–4EE, long-range EE, and, for the first time, Generalized Born contributions) as well as the angular and dihedral energies. Some deviations with respect to the CMD counterpart are expected due to the multiple protonation states over time. The deprotonated systems (ASP^CMD^ and AS4^CPHMD^ at pH 12) present similar total and potential energies, but the same behavior is reproduced in the electrostatics, angular, and dihedral contributions. In fact, the shift of the distribution is more pronounced in the electrostatics interactions. The angular and dihedral terms of the AS4^CPHMD^ systems at acid and basic conditions greatly overlap between them, excluding their counterparts (ASH^CMD^ and ASP^CMD^). The electrostatics decomposition into backbone and side chain atoms (Figure 10) evidence that the latter contribution caused the divergence in the electrostatics for the protonated simulations. This fact is probably related with the change of protonation states (and partial charges in the atoms) during the simulation. The two peaks shown in the side chain electrostatics in the AS4^CPHMD^ at pH 1 correspond to the syn-O1 and syn-O2 protonation in their most stable conformation. For the deprotonated systems, we observed a mismatch of the distributions in both side chain and backbone contributions. It is easily explained by the different partial charges in the backbone atoms, while the shift on the side chain is caused by the partial charge of the C_β_ atom.

The energy distributions of the GLU systems show similar results as the ASP amino acid. The protonated simulations (GLH^CMD^ and GL4^CPHMD^ at pH 1) show dissimilarities in the kinetic and potential energies, concretely in the angular, dihedral, and electrostatic terms (Figure S13). However, the differences in electrostatic energy are smaller than those observed in the ASP systems given that the distributions are in the same energy range, but shapes do not coincide. On the contrary, the distributions of the deprotonated systems (GLU^CMD^ and GL4^CPHMD^ at pH 12) have a greater shift for the total, potential, and 1–4 electrostatics terms, and a similar energy range for the long-range electrostatics. The decomposition of the electrostatics terms (Figure S14) shows that backbone atoms reproduce correctly the electrostatics interactions for the protonated systems (GLH^CMD^ and GL4^CPHMD^ at pH 1). The electrostatics of the side chain atoms are not equal, which we associate with the multiple protonation state. The distributions of the deprotonated systems (GLU^CMD^ and GL4^CPHMD^ at pH 12) evidence differences in the backbone and side chain contributions for both electrostatics terms. The shapes in the electrostatics of the side chain suggest different protonation state sampling in the CPHMD simulations with respect to the CMD counterparts.

Some deficiencies are identified in the analysis of the energy distributions. An accurate description of the electrostatics is crucial to ensure the reproducibility of the simulation and, therefore, obtain a good conformational sampling. The energy decomposition allowed clarifying some points. On one hand, the backbone electrostatics shows that protonated simulations are in agreement, while the deprotonated ones do not. As it was explained in the previous section, the partial charge of the backbone atoms in the CPHMD method are fixed in those of the protonated state, leading to an inaccurate description of the electrostatic interactions when the residue is deprotonated. Thus, deprotonated CPHMD simulations cannot reproduce the electrostatic distributions of the CMD counterpart. In fact, the backbone electrostatics of deprotonated CPHMD systems usually overlap with the protonated distributions. Smooth differences may be observed due to the different conformational sampling. In particular, the CYS systems could be disputed since they have a correct *global* electrostatics distribution, but if the backbone and side chain contributions are considered separately, the CMD and CPHMD counterparts do not have similar distributions. On the other hand, the side chain electrostatics generally shows discrepancies in the deprotonated simulations of all amino acids and the protonated simulations of HIS and acidic amino acids. This alteration is caused by two factors: (i) the modified partial charge of the C_β_ atom to ensure a net charge change of ±1.0, which affected the distributions of the deprotonated forms, and (ii) the amino acids with multiple protonatable sites in the CPHMD method are not comparable to the CMD counterparts given that the partial charges of the side chain atoms of the CPHMD residues vary during the simulation. This is perceived in the deprotonated form of HIS and the protonated ones of the acidic amino acids, including the Generalized Born electrostatics of the last set.

The energy distributions also suggest that angular and dihedral energies are not properly described in these multiple protonatable amino acids. It seems plausible that the divergence in these two terms is not due to the partial charges and, instead, it could be caused by (i) the activation and deactivation of the hydrogen during the protonation change and/or (ii) the building of the CPHMD residues since hydrogens are present in the simulation as ghost atoms.

### 3.3. Side Chain Orientation and Atom Distances

**Figure 11:**
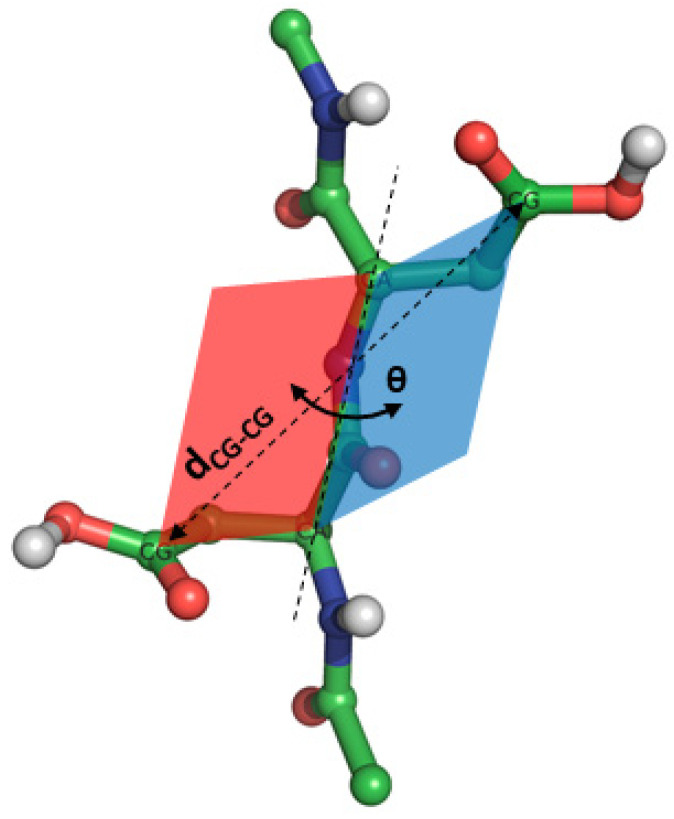
Dihedral angle (θ) constructed using the alpha carbon (CA) atoms and two selected atoms located in the side chain. In this case, carboxyl carbon (CG) atoms are selected. Table S3 indicates the atom selection for each amino acid.

**Figure 12:**
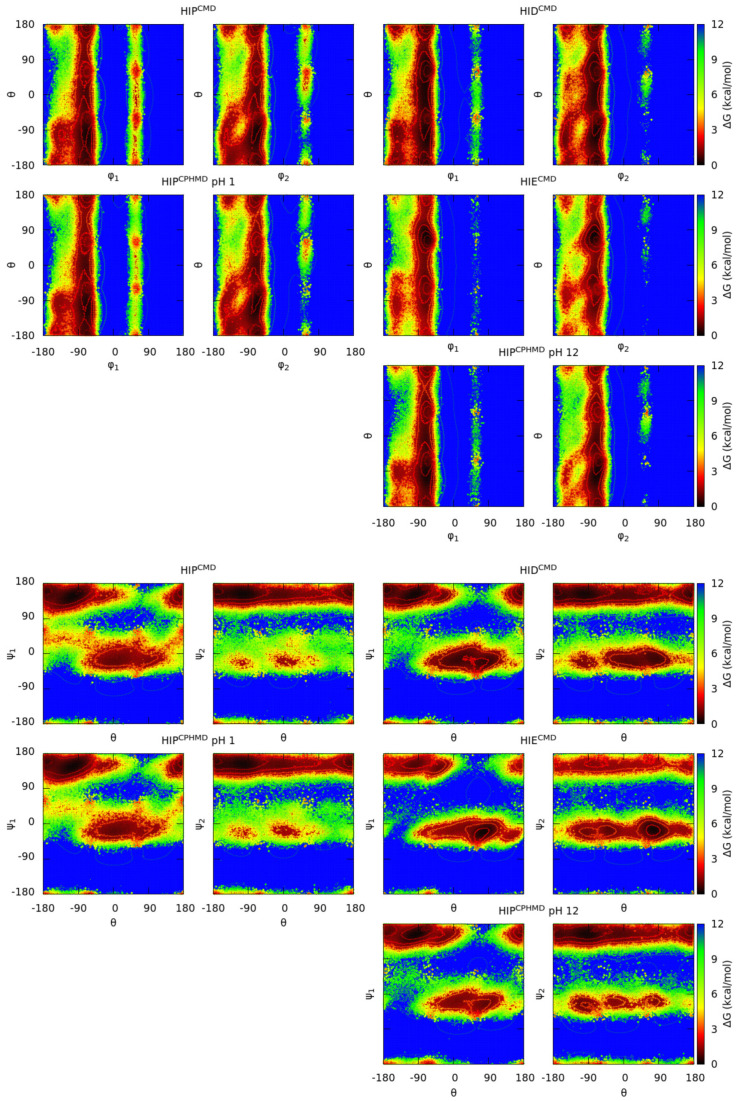
Gibbs free energies in the side chain-orientation space of the blocked His_2_ tripeptide. Each subtitle indicates the residue, the simulation method (in the superscript), and the pH (only for the CPHMD simulations). Four sets of dihedral angles are used in this plot, using the θ dihedral angle (CE1-CA-CA-CE1) in conjunction with the phi (φ) or psi (ψ) of each monomer (φ_1_/ψ_1_ from the N-terminal amino acid; φ_2_/ψ_2_ from the C-terminal amino acid). Protonated forms are on the left and deprotonated ones are on the right. Solid lines indicate an increase of 0.6 kcal/mol of the energy values.

Finally, the φ/ψ dihedral angles and the characteristic dihedral, which is constructed by the main chain C_α_ atom and a selected side chain atom of each amino acid, were used to define a new representation of conformational space. This dihedral, called angle θ, is more suitable to provide knowledge of the orientation of the side chains with respect to the backbone. Then, the side chain-orientation space is divided into four sets: the φ_i_/θ and θ/ψ_i_, where i is the N-terminal (monomer 1) or C-terminal (monomer 2) amino acid. Figure 11 illustrates the θ dihedral angle, and Table S3 reports the selected atoms for the θ angle for each amino acid. The map of the blocked HIS_2_ tripeptide is illustrated in Figure 12, and the others are illustrated in Supplementary Materials (Figures S15–S19). The distribution of this interatomic distance between the selected atoms is plotted in Figure S21.

In general, deprotonated and protonated simulations are in line with the results of the Ramachandran maps. Protonated systems of all amino acids, except for GLU and ASP, show a good concordance of the conformational sampling as well as the distances of the specific atoms. In contrast, the GLU and ASP systems present mild differences in both conformational sampling and atomic distances, which is on basis of previous results. For all the amino acids, the conformational sampling of the deprotonated forms diverges between the CMD and CPHMD counterparts, being less noticeable for CYS and LYS and more significant for HIS, GLU and ASP.

This subsection (Section 3.3) corroborates the results of Ramachandran maps and energy distribution. However, the definition of this new angle and the construction of these maps (in the φ/θ and θ/ψ space) provide new information about the HIP^CPHMD^ at pH 12. The atomic distances and the plots are similar to the HID^CMD^ system rather than HIE^CMD^, which seems plausible, since the side chain electrostatics of HIP^CPHMD^ at pH 12 are closer to the HID^CMD^. In fact, this conclusion is in agreement with the population of the delta state during the CPHMD simulation (77% and 81% for monomer 1 and 2, respectively) in contrast to the epsilon state (23% and 19%). On the other hand, the GLU and ASP residues present another behavior. In these systems, the plots show that CMD and CPHMD counterparts (e.g., in the case of GLU, the GLH^CMD^ and GL4^CPHMD^ at pH 1 systems for the protonated form, and the GLU^CMD^ and GL4^CPHMD^ at pH 12 systems for the deprotonated one) have a similar conformational sampling, even though closer atomic distances are shown when using the same simulation method. Despite the difference in the atomic distance is small, it may be caused by a lack of description of angle and dihedral energies.

## 4. Conclusions

Ramachandran maps and energy distributions have shown that the CPHMD method can reproduce the conformational sampling of the protonated forms of the tripeptides simulated with the CMD method. For the deprotonated forms, the different assignment of the partial charges of the backbone atoms in the AMBER implementation leads to inaccuracies in the conformational profiles and in the energy distributions with respect to the CMD simulations. The electrostatic distributions show good agreement in the protonated forms, while deprotonated ones present significant changes. The decomposition of the energy into backbone and side chain contributions exhibit that backbone electrostatics of the protonated form (the reference state) for CMD simulations and CPHMD simulations have similar distributions. Instead, the deprotonated CMD systems present a different distribution. The mismatch in energy between deprotonated forms and the incorrect overlapping of the energy distribution of the deprotonated CPHMD system with the distributions of the protonated forms is due to fixing the partial charges of the backbone atoms during the simulation. For the side chain electrostatics, mild differences are observed in the deprotonated forms due to the modified partial charge of the C_β_ atom as well as the protonated forms of the acidic amino acids. The acidic amino acids do not perfectly overlap in the side chain electrostatics due to the multiple protonatable sites in the CPHMD simulations, showing an energy distribution with two peaks that correspond to the protonation in the *syn* position of each oxygen atom. Moreover, these amino acids that can be protonated in distinct sites, in which deprotonated HIS^CPHMD^ is also included, show disagreement in the description of the angular and dihedral energies. Due to the different protonation state sampling in the two simulation methods, the Ramachandran maps and the energy distributions of these residues are not fully comparable. Thus, the change of protonation states may suppose an advantage for the conformational sampling rather than being considered an incorrect description of the amino acids.

The CPHMD method represents an improvement in the simulation of biomolecular systems. In principle, the protonation state sampling that CPHMD methods provide allows a better description of the protonation state (and, therefore, the conformational sampling) according to the chemical environment and the time evolution of the system. For amino acids that have more than one protonation state in the protonated form, the mobility of the protons can provide a better description rather than CMD simulations. However, the results extracted from the Ramachandran maps expose a deficiency in the conformational sampling of the deprotonated forms due to the fixed partial charges of the backbone atoms. Therefore, we recommend the use of the CPHMD method in the AMBER implementation with caution, given that the effects of the incorrect partial charges in the backbone atoms are unknown, and comparing structural protein descriptors (R_g_, chemical shifts, FRET measurements…) with experimental data whenever possible.

## Figures and Tables

**Table 1:** Simulations performed per each residue type and methodology. Protonation state is defined as deprotonated (D), protonated (P) or titratable (T) form. Some residues can be used to build protonated conventional Molecular Dynamics (CMD) simulations as well as titratable Constant pH Molecular Dynamics (CPHMD) systems. AMBER’s intrinsic pKa values are used according to Mongan et al. [27].

Residue	CMD	CpHMD			Prot. State.	Intrinsic pKa
		pH 1	pH 12	pH 14		
ASP	✓				D	4.0
ASH	✓				P	
AS4		✓	✓		T	
GLU	✓				D	4.4
GLH	✓				P	
GL4		✓	✓		T	
HIE	✓				D	7.1 (ε) 6.5 (δ)
HID	✓				D	
HIP	✓	✓	✓		P/T	
CYM	✓				D	8.5
CYS	✓	✓	✓		P/T	
TYR	✓	✓			P/T	9.6
LYN	✓				D	10.4
LYS	✓	✓		✓	P/T	

## Data Availability

Data available on request due to restrictions e.g., privacy or ethical.

## Supplementary Materials

The following are available online at https://www.mdpi.com/2073-4360/13/1/99/s1, Figure S1: Protonatable sites in the side chain of the aspartic acid, Figure S2: Classification of the nine secondary structure regions (C_5_, P_II_, α_D_, β2, C_7_^axial^, α_L_, α’, α_R_ and C_7_^eq^) in the Ramachandran space by J. Rubio-Martinez et al., Figure S3: Gibbs free energies in the Ramachandran space of the blocked Tyr_2_ tripeptide, Figure S4: Gibbs free energies in the Ramachandran space of the blocked Lys_2_ tripeptide, Figure S5: Gibbs free energies in the Ramachandran space of the blocked His_2_ tripeptide, Figure S6: Gibbs free energies in the Ramachandran space of the blocked Glu_2_ tripeptide, Figure S7: Energy distributions of the blocked Tyr_2_ tripeptide, Figure S8: Energy distributions of the blocked Cys_2_ tripeptide, Figure S9: Energy distribution of the 1–4 and long-range electrostatics of the backbone and side chain atoms of the blocked Tyr_2_ tripeptide, Figure S10: Energy distribution of the 1–4 and long-range electrostatics of the backbone and side chain atoms of the blocked Cys_2_ tripeptide, Figure S11: Energy distributions of the blocked His_2_ tripeptide, Figure S12: Energy distribution of the 1–4 and long-range electrostatics of the backbone and side chain atoms of the blocked His_2_ tripeptide, Figure S13: Energy distributions of the blocked Glu_2_ tripeptide, Figure S14: Energy distribution of the 1–4 and long-range electrostatics of the backbone and side chain atoms of the blocked Glu_2_ tripeptide, Figure S15: Gibbs free energies in the side chain-orientation space of the blocked Lys_2_ tripeptide, Figure S16: Gibbs free energies in the side chain-orientation space of the blocked Tyr_2_ tripeptide, Figure S17: Gibbs free energies in the side chain-orientation space of the blocked Cys_2_ tripeptide, Figure S18: Gibbs free energies in the side chain-orientation space of the blocked Glu_2_ tripeptide, Figure S19: Gibbs free energies in the side chain-orientation space of the blocked Asp_2_ tripeptide, Figure S20: Distribution of the atomic distance between the atoms of the side chain selected for the construction of the θ dihedral angle, Table S1: Partial charges of the protonated and deprotonated forms of the Glu and Asp amino acids in CMD and CPHMD simulations, Table S2: Partial charges of the protonated and deprotonated forms of the Lys, Tyr, Cys and His amino acids in CMD and CPHMD simulations, Table S3: Averages and standard deviations of the interatomic distance using the selected atoms at the extreme of the side chains.
