# Supplementary material for: On the Use of the Discrete Constant pH Molecular Dynamics to Describe the Conformational Space of Peptides
Source: Polymers (Basel). 2020 Dec 29;13(1):99. doi: 10.3390/polym13010099 (PMC7795291; doi:10.3390/polym13010099)
Supplement: Supplementary file 1 [file polymers-13-00099-s001.pdf]

Supporting Information: On the use of the Discrete Constant pH Molecular Dynamics to describe the Conformational Space of Peptides

Cristian Privat [1], Sergio Madurga [1,*], Francesc Mas [1] and Jaime Rubio-Martínez [1,*]

[1] Department of Material Science and Physical Chemistry & Research Institute of Theoretical and Computational Chemistry (IQTCUB), University of Barcelona, C/ Martí i Franquès 1, 08028, Barcelona, Spain

* Correspondence: jaime.rubio@ub.edu (J.R-M.); s.madurga@ub.edu (S.M.)

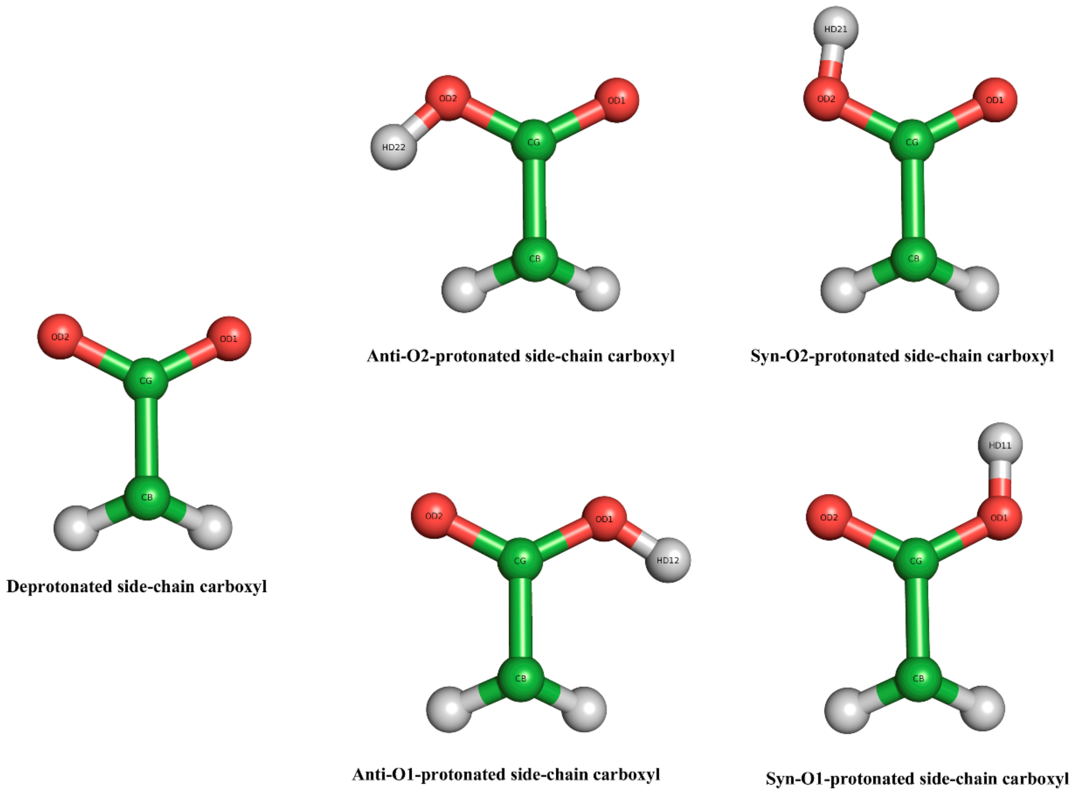

Figure S1. Protonatable sites in the side chain of the aspartic acid. There are four protonatable sites that correspond to the anti- or syn- position with respect to each oxygen of the carboxyl group. CPHMD method builds a residue with the four hydrogens, and only make one or none of them *effective* according to the protonation state.

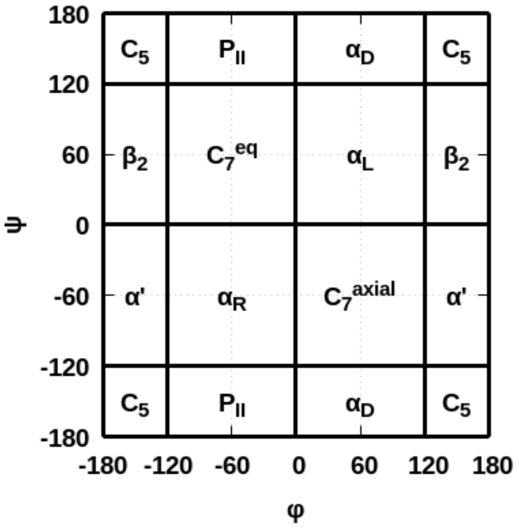

Figure S2. Classification of the nine secondary structure regions (C_5, P_{II}, α_D, β_2, C_7^{eq}, α_L, α', α_R and C_7^{axial}) in the Ramachandran space by J. Rubio-Martinez et al.[1].

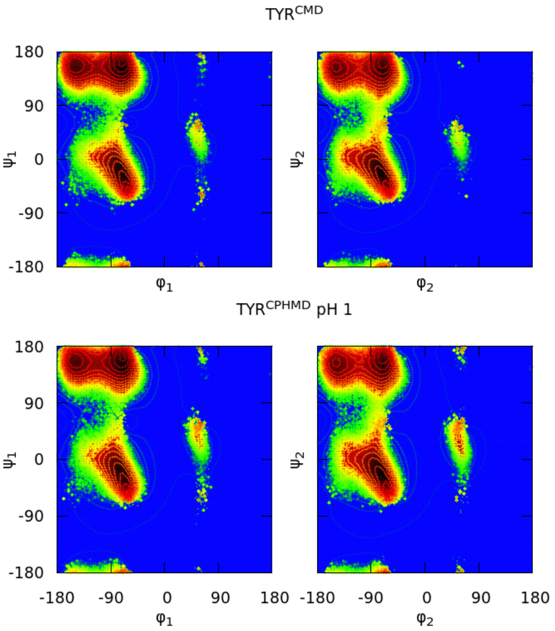

Figure S3. Gibbs free energies in the Ramachandran space of the blocked Tyr_2 tripeptide. The labelling indicates the residue, the simulation method (in the superscript) and the pH (only for the CPHMD simulations). Both sets of dihedrals (φ_1/ψ_1 from the N-terminal amino acid; φ_2/ψ_2 from the C-terminal amino acid) are illustrated. Protonated forms are in the left (CMD; top – CPHMD; bottom) and deprotonated ones in the right (CMD; top – CPHMD; bottom). Solid lines indicate an increase of 0.6 kcal/mol of the energy values.

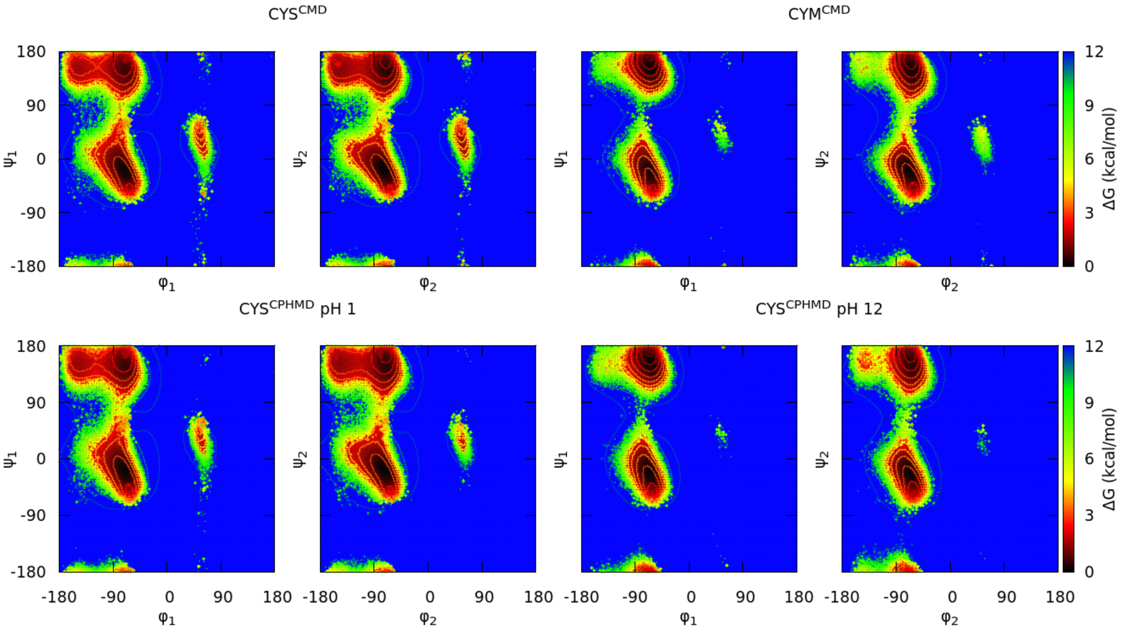

Figure S4. Gibbs free energies in the Ramachandran space of the blocked Cys_2 tripeptide. The labelling indicates the residue, the simulation method (in the superscript) and the pH (only for the CPHMD simulations). Both sets of dihedrals (φ_1/ψ_1 from the N-terminal amino acid; φ_2/ψ_2 from the C-terminal amino acid) are illustrated. Protonated forms are in the left (CMD; top – CPHMD; bottom) and deprotonated ones in the right (CMD; top – CPHMD; bottom). Solid lines indicate an increase of 0.6 kcal/mol of the energy values.

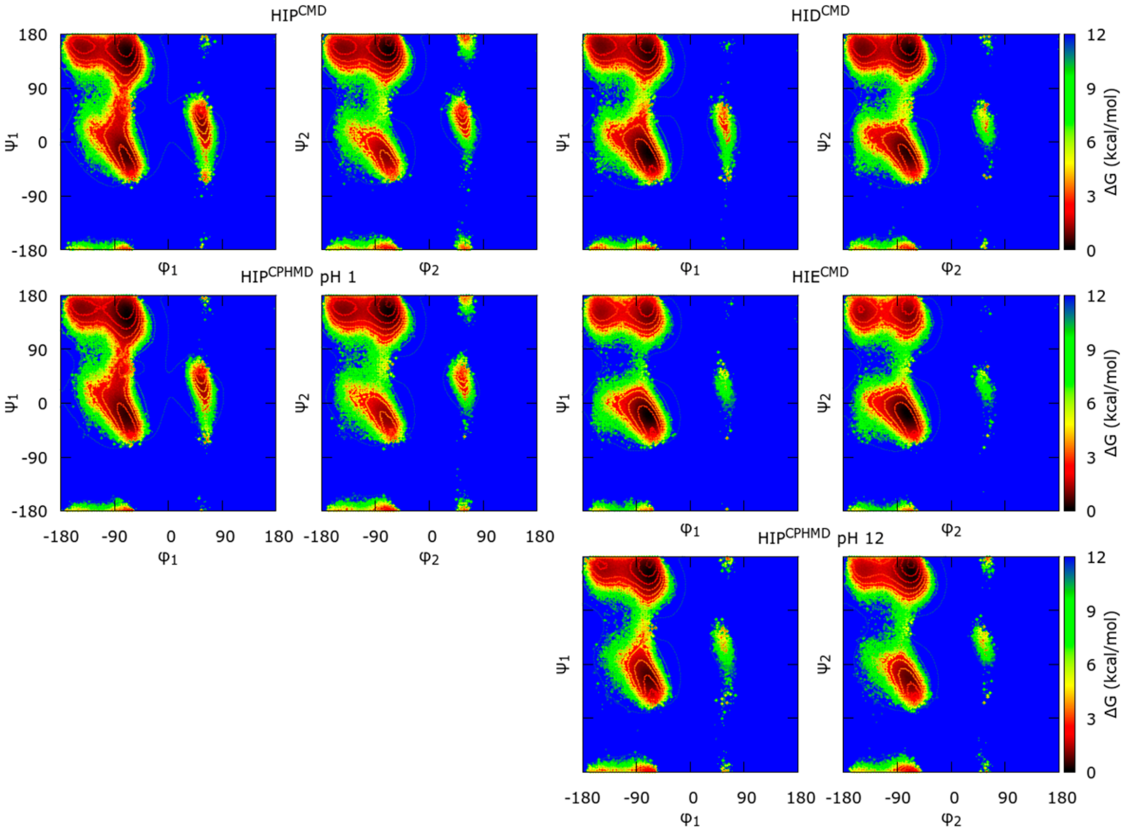

Figure S5. Gibbs free energies in the Ramachandran space of the blocked His$_2$ tripeptide. The labelling indicates the residue, the simulation method (in the superscript) and the pH (only for the CPHMD simulations). Both sets of dihedrals (φ_1/ψ_1 from the N-terminal amino acid; φ_2/ψ_2 from the C-terminal amino acid) are illustrated. Protonated forms are in the left (CMD; top – CPHMD; bottom) and deprotonated ones in the right (CMD; top – CPHMD; bottom). Solid lines indicate an increase of 0.6 kcal/mol of the energy values.

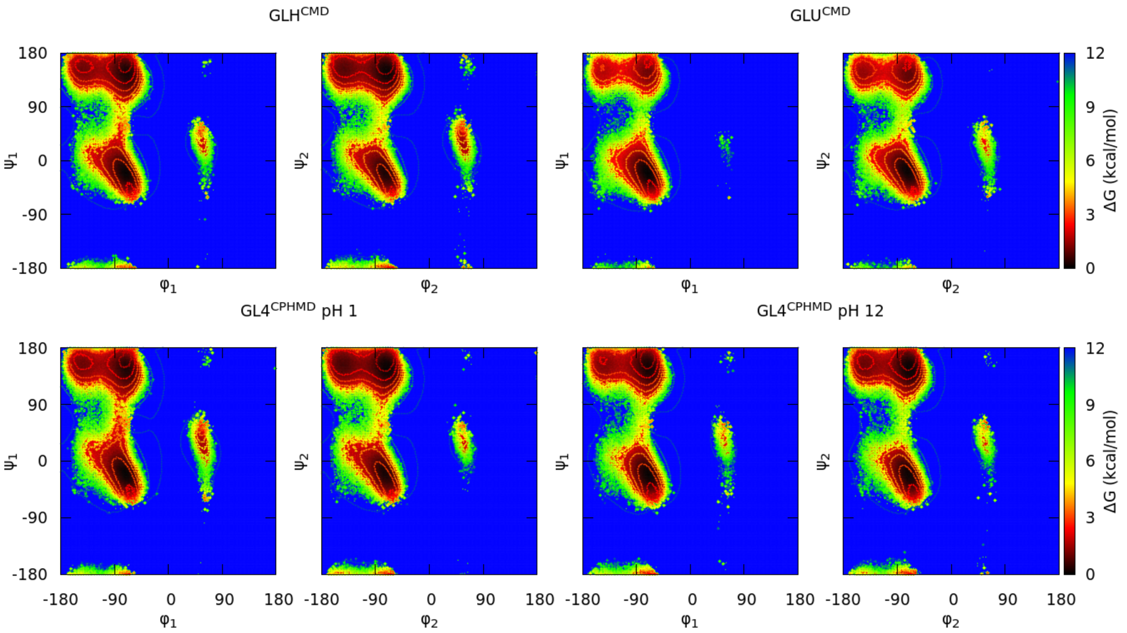

Figure S6. Gibbs free energies in the Ramachandran space of the blocked Glu$_2$ tripeptide. The labelling indicates the residue, the simulation method (in the superscript) and the pH (only for the CPHMD simulations). Both sets of dihedrals (φ_1/ψ_1 from the N-terminal amino acid; φ_2/ψ_2 from the C-terminal amino acid) are illustrated. Protonated forms are in the left (CMD; top – CPHMD; bottom) and deprotonated ones in the right (CMD; top – CPHMD; bottom). Solid lines indicate an increase of 0.6 kcal/mol of the energy values.

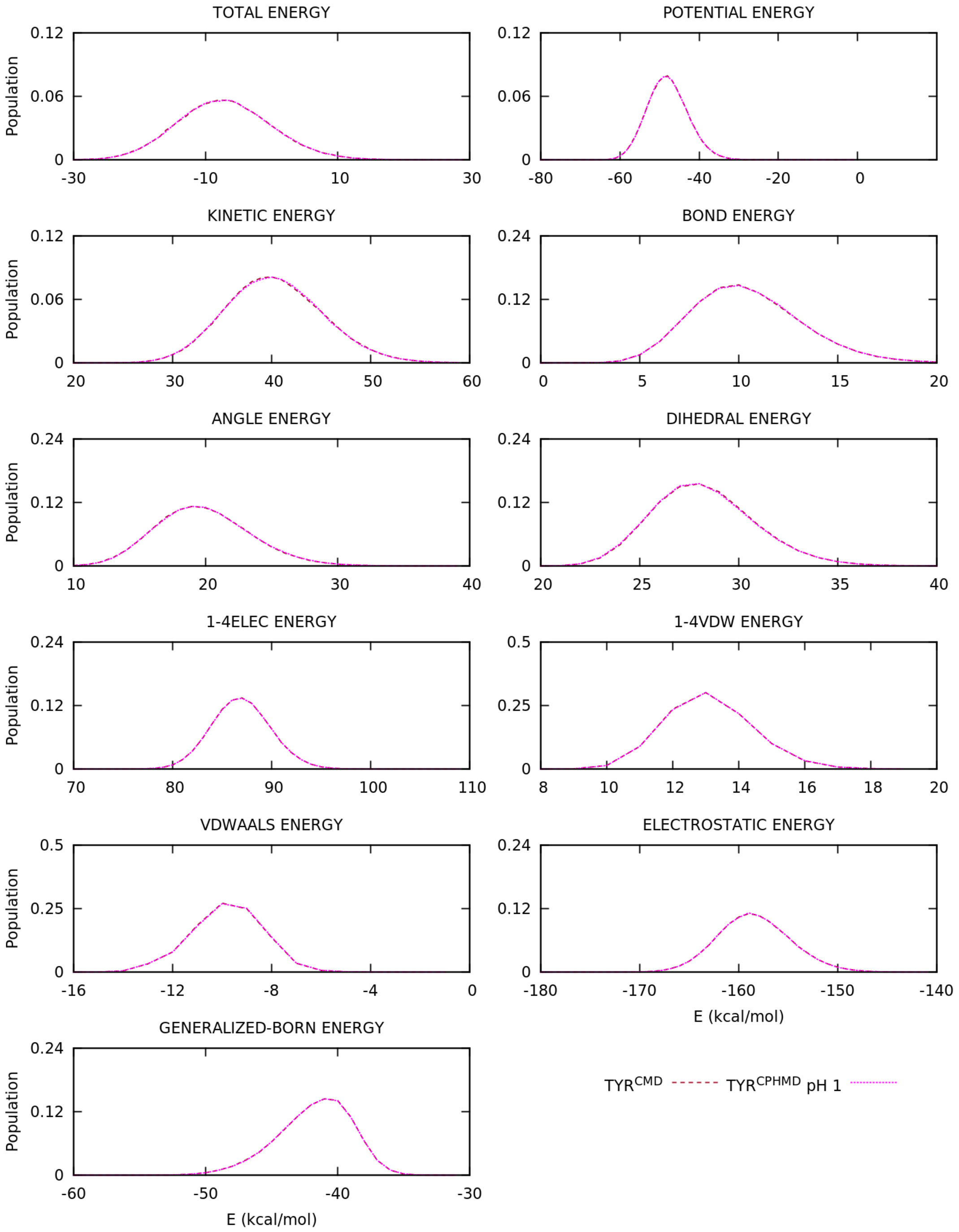

Figure S7. Energy distributions of the blocked Tyr$_2$ tripeptide. Global, inner, van der Waals and electrostatics terms are illustrated. Dotted and dashed lines are CPHMD and CMD simulation methods, respectively.

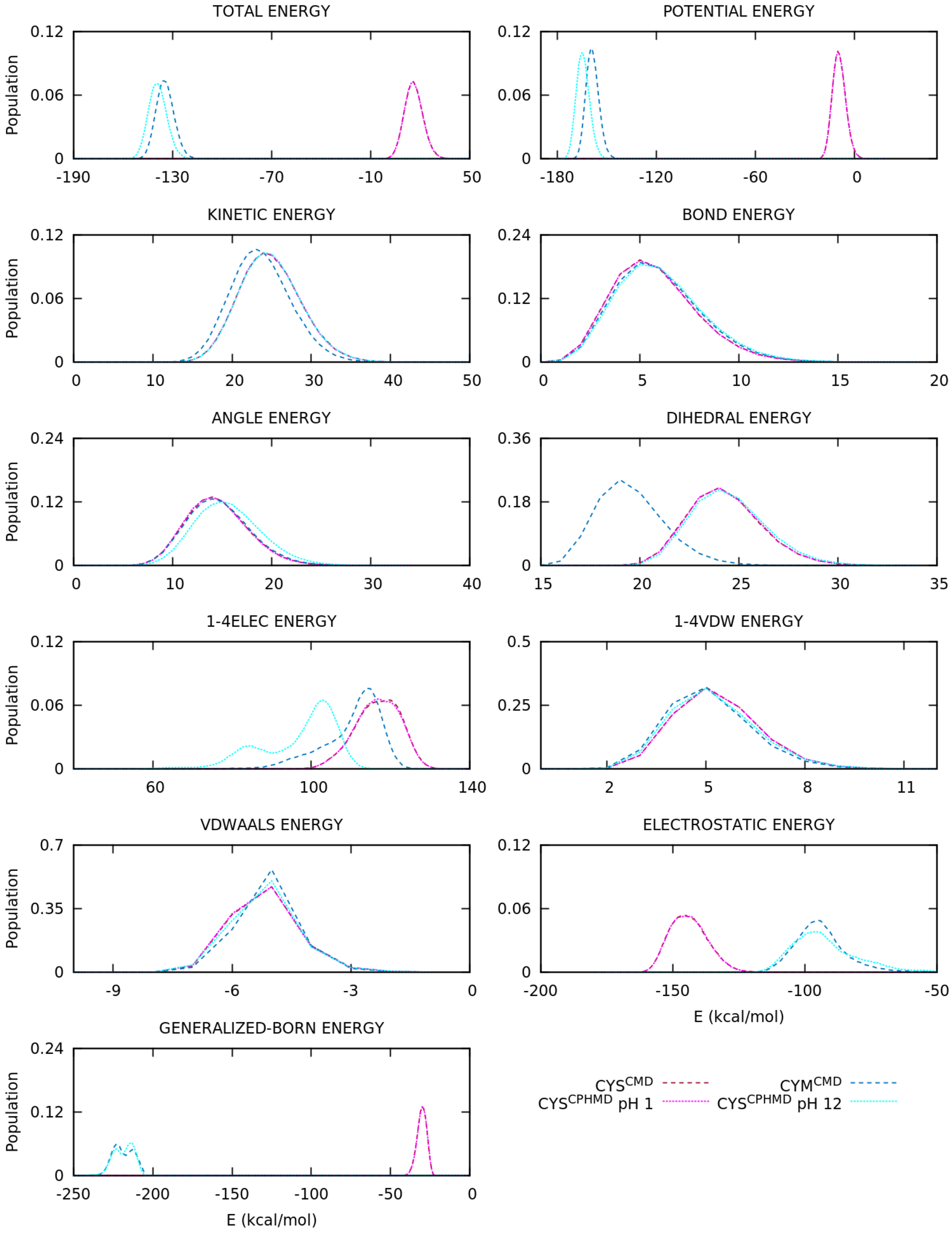

Figure S8. Energy distributions of the blocked Cys2 tripeptide. Global, inner, van der Waals and electrostatics terms are illustrated. Dotted and dashed lines are CPHMD and CMD simulation methods, respectively.

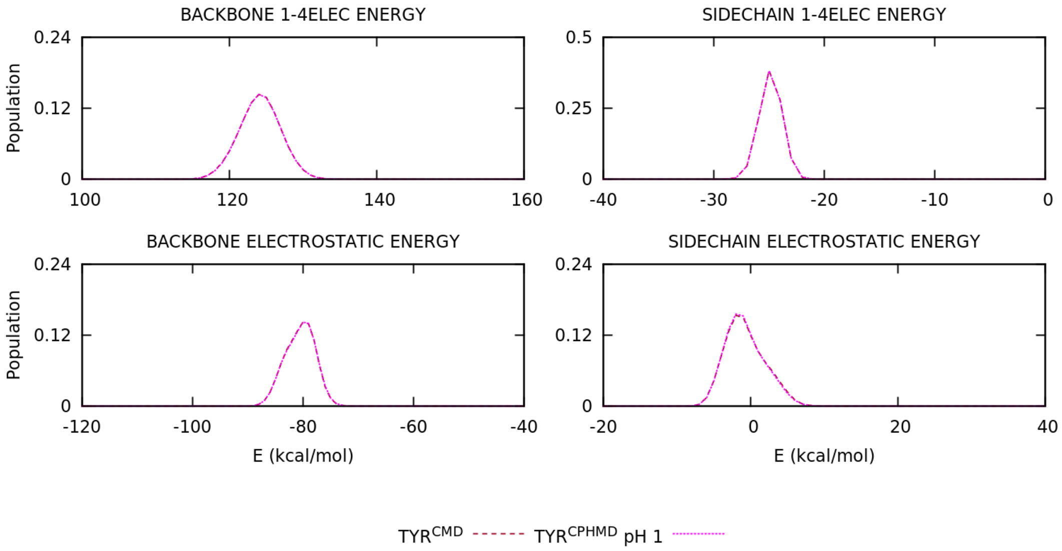

Figure S9. Energy distribution of the 1-4 and long-range electrostatics of the backbone and sidechain atoms of the blocked Tyr2 tripeptide. Dotted and dashed lines are CPHMD and CMD simulation methods, respectively.

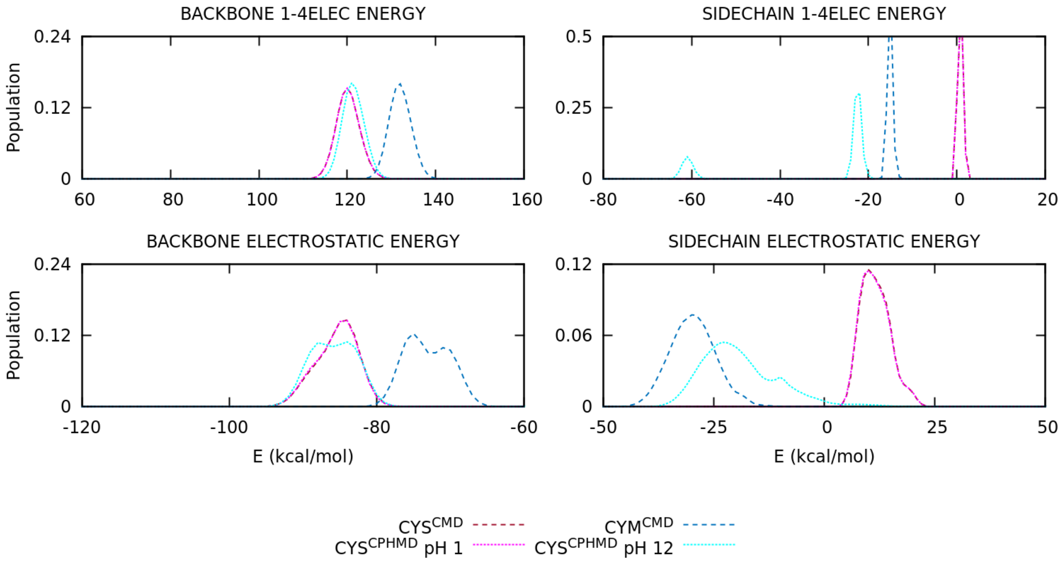

Figure S10. Energy distribution of the 1-4 and long-range electrostatics of the backbone and sidechain atoms of the blocked Tyr2 tripeptide. Dotted and dashed lines are CPHMD and CMD simulation methods, respectively.

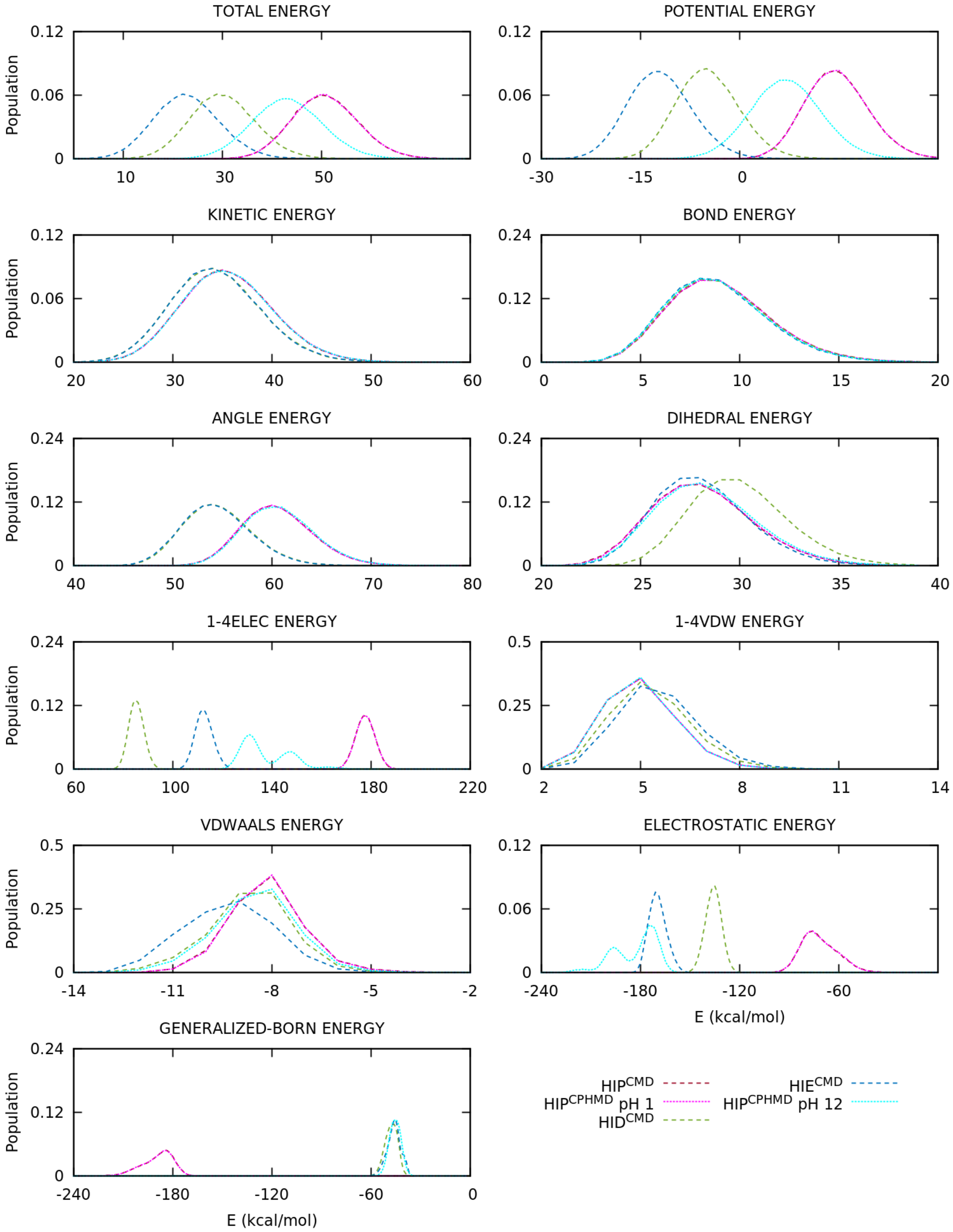

Figure S11. Energy distributions of the blocked His_2 tripeptide. Global, inner, van der Waals and electrostatics terms are illustrated. Dotted and dashed lines are CPHMD and CMD simulation methods, respectively.

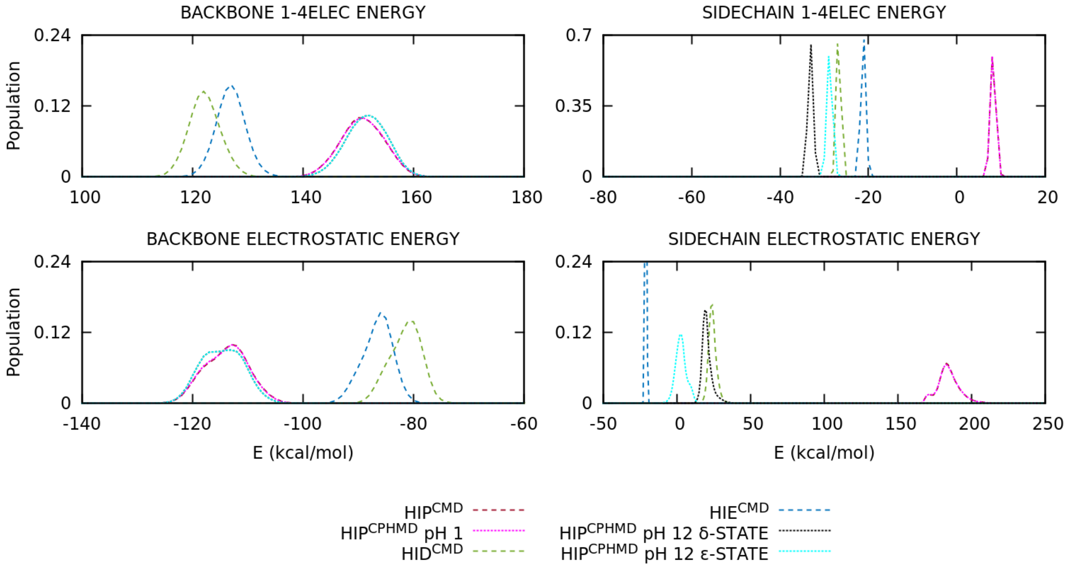

Figure S12. Energy distribution of the 1-4 and long-range electrostatics of the backbone and sidechain atoms of the blocked His_2 tripeptide. Dotted and dashed lines are CPHMD and CMD simulation methods, respectively. Labels δ- and ε-STATE refer to the partial charges used for the computation of the side chain electrostatic energies.

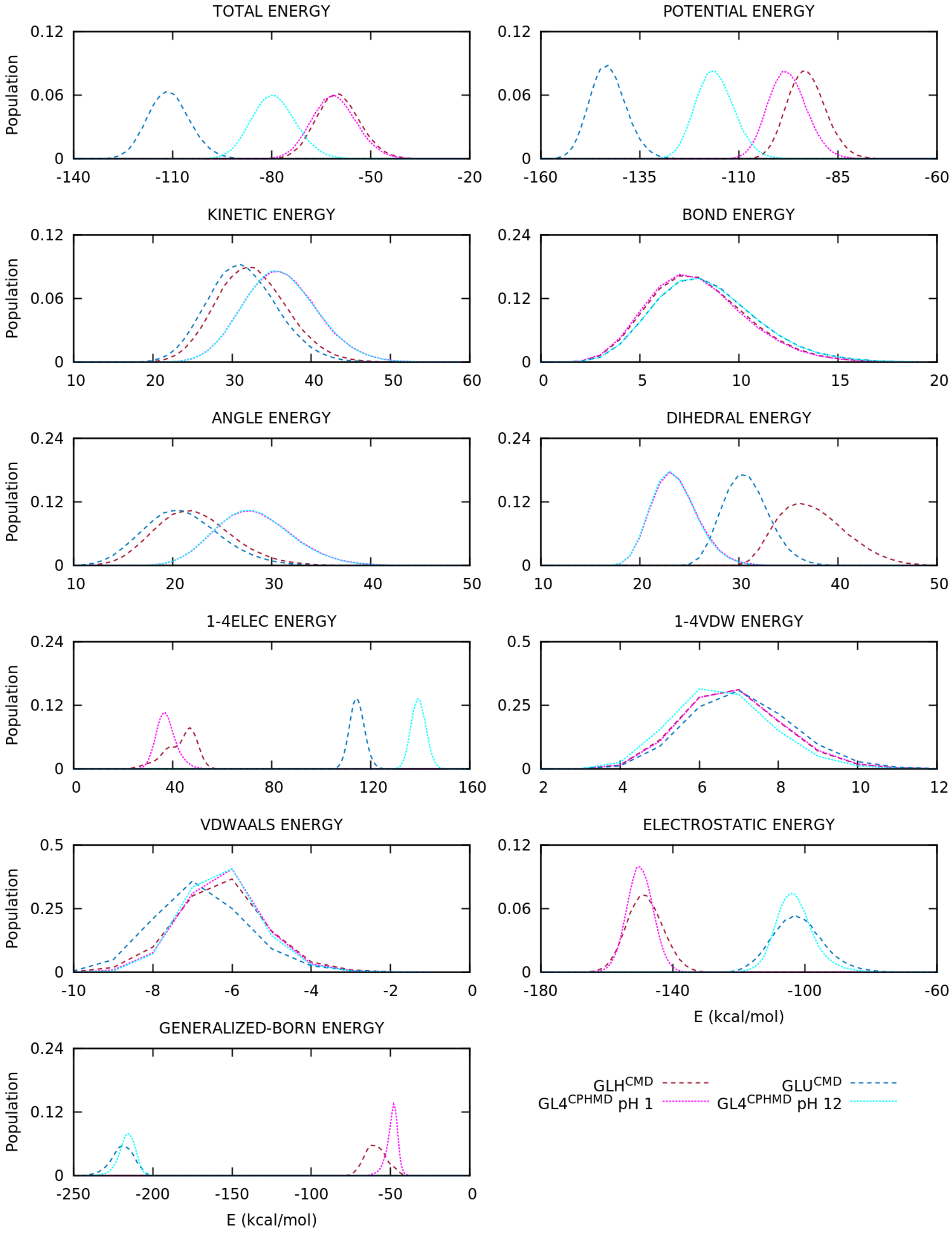

Figure S13. Energy distributions of the blocked Glu_2 tripeptide. Global, inner, van der Waals and electrostatics terms are illustrated. Dotted and dashed lines are CPHMD and CMD simulation methods, respectively.

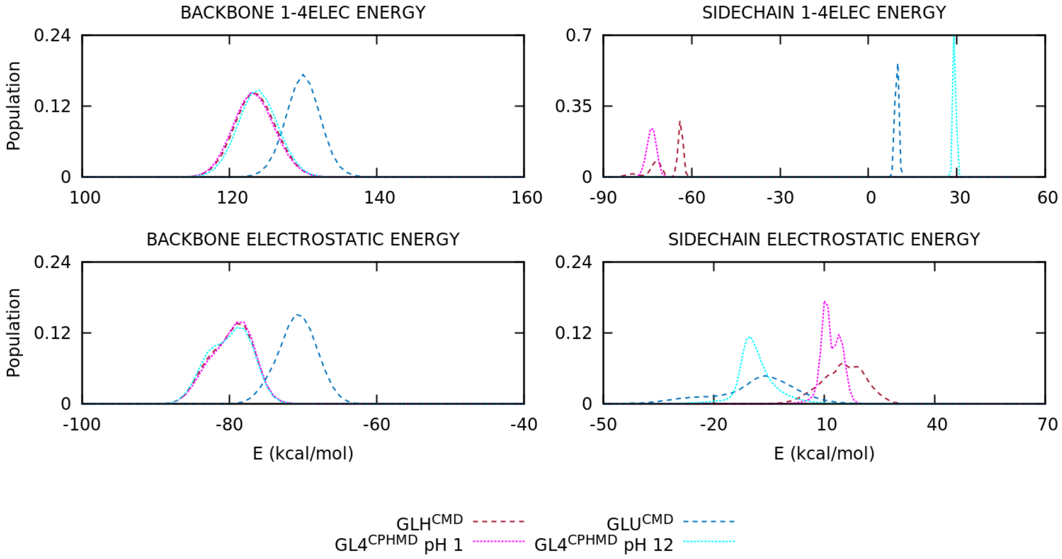

Figure S14. Energy distribution of the 1-4 and long-range electrostatics of the backbone and sidechain atoms of the blocked Glu_2 tripeptide. Dotted and dashed lines are CPHMD and CMD simulation methods, respectively.

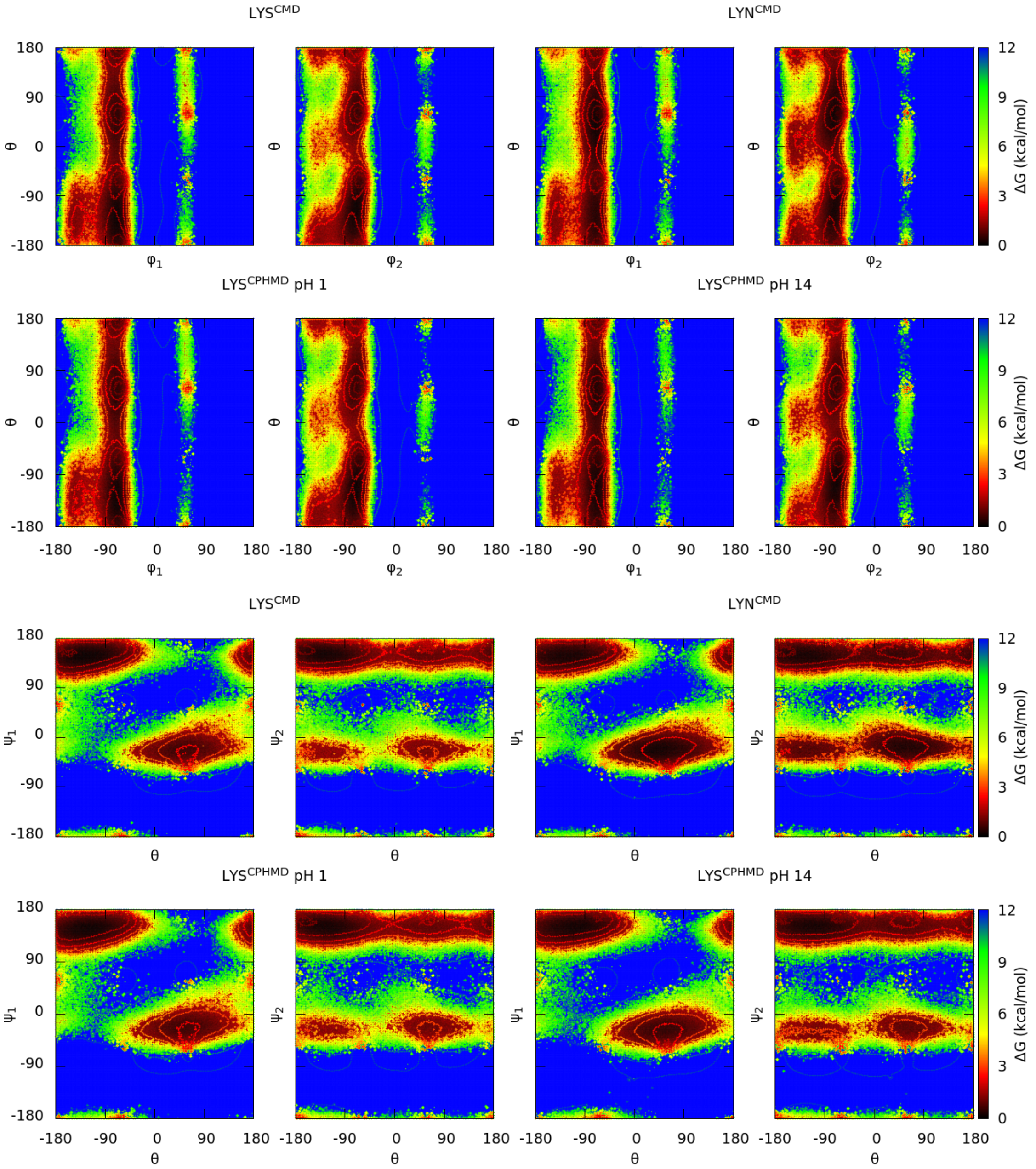

Figure S15. Gibbs free energies in the sidechain-orientation space of the blocked Lys_2 tripeptide. The labelling indicates the residue, the simulation method (in the superscript) and the pH (only for the CPHMD simulations). Four sets of dihedral angles are used in this plot, using the θ dihedral angle (CE1-CA-CA-CE1) in conjunction with the phi (φ) or psi (ψ) of each monomer (φ_1/ψ_1 from N-terminal amino acid; φ_2/ψ_2 from the C-terminal amino acid). Protonated forms are in the left and deprotonated ones in the right. Solid lines indicate an increase of 0.6 kcal/mol of the energy values.

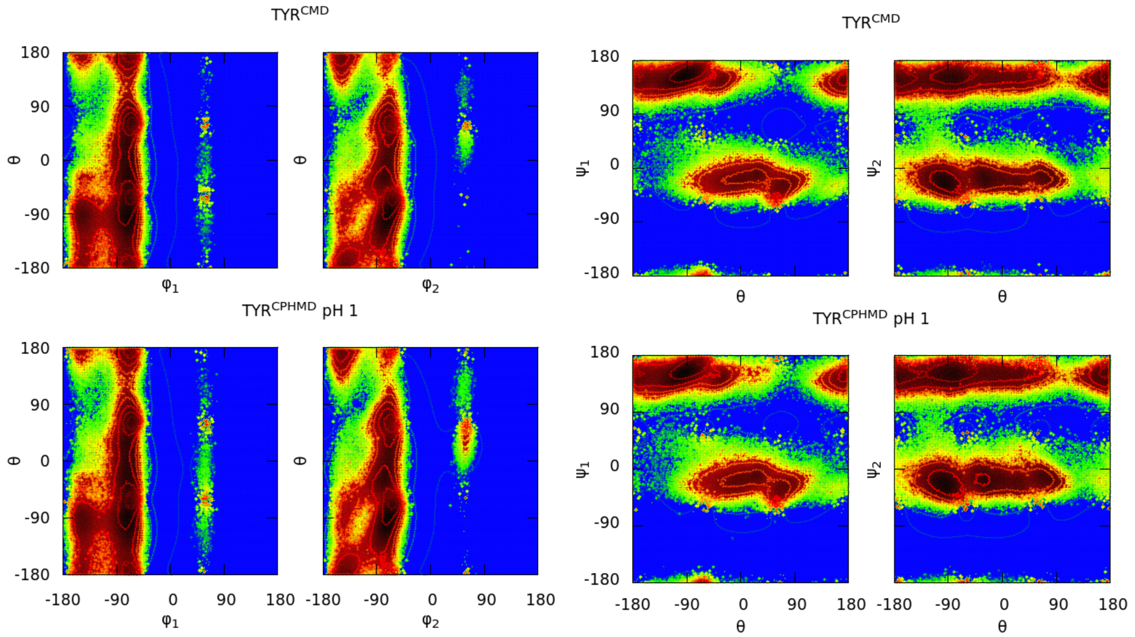

Figure S16. Gibbs free energies in the sidechain-orientation space of the blocked Tyr2 tripeptide. The labelling indicates the residue, the simulation method (in the superscript) and the pH (only for the CPHMD simulations). Four sets of dihedral angles are used in this plot, using the θ dihedral angle (CE1-CA-CA-CE1) in conjunction with the phi (φ) or psi (ψ) of each monomer (φ_1/ψ_1 from N-terminal amino acid; φ_2/ψ_2 from the C-terminal amino acid). Protonated forms are in the left and deprotonated ones in the right. Solid lines indicate an increase of 0.6 kcal/mol of the energy values.

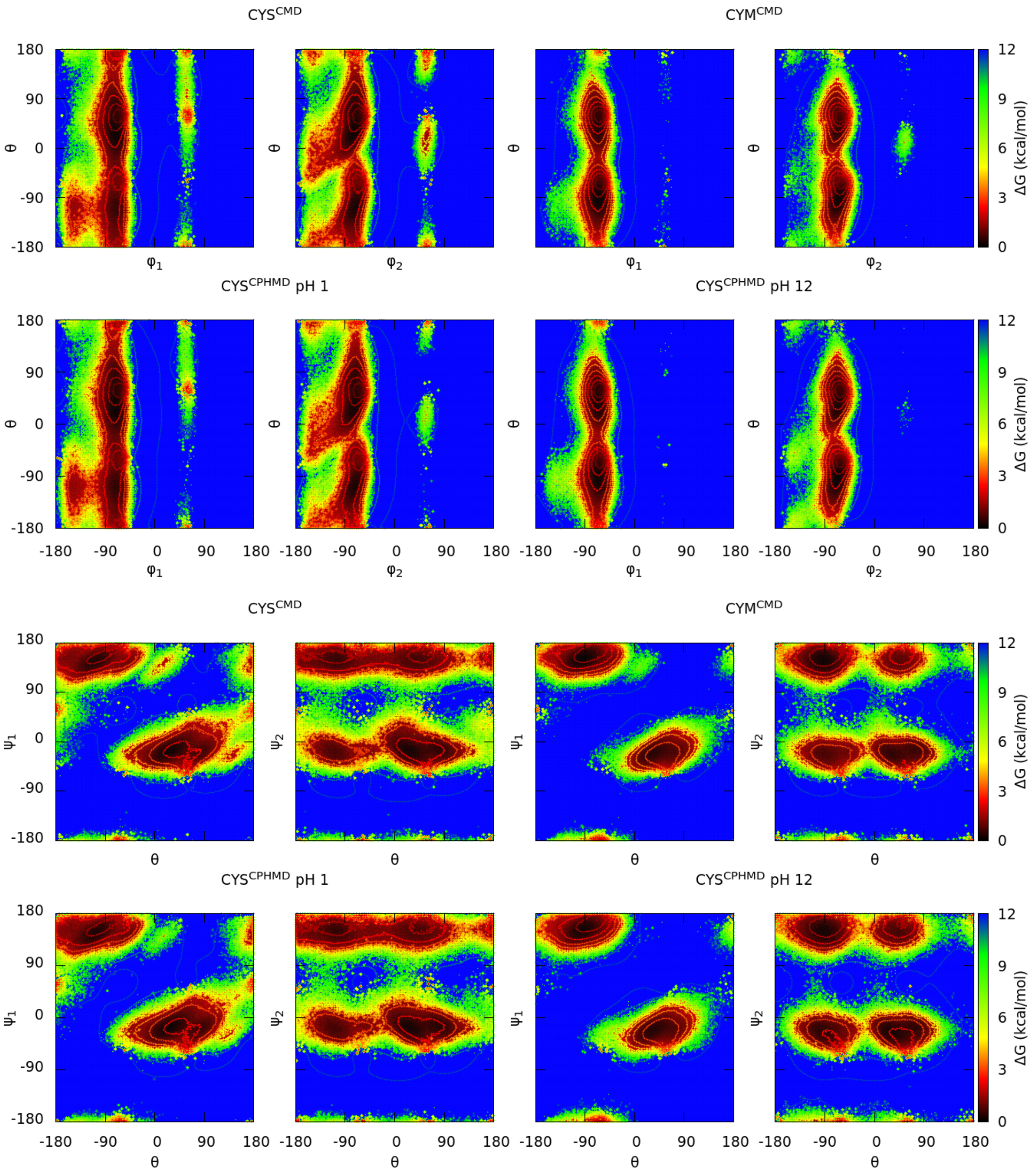

Figure S17. Gibbs free energies in the sidechain-orientation space of the blocked Cys$_2$ tripeptide. The labelling indicates the residue, the simulation method (in the superscript) and the pH (only for the CPHMD simulations). Four sets of dihedral angles are used in this plot, using the θ dihedral angle (CE1-CA-CA-CE1) in conjunction with the phi (φ) or psi (ψ) of each monomer (φ_1/ψ_1 from N-terminal amino acid; φ_2/ψ_2 from the C-terminal amino acid). Protonated forms are in the left and deprotonated ones in the right. Solid lines indicate an increase of 0.6 kcal/mol of the energy values.

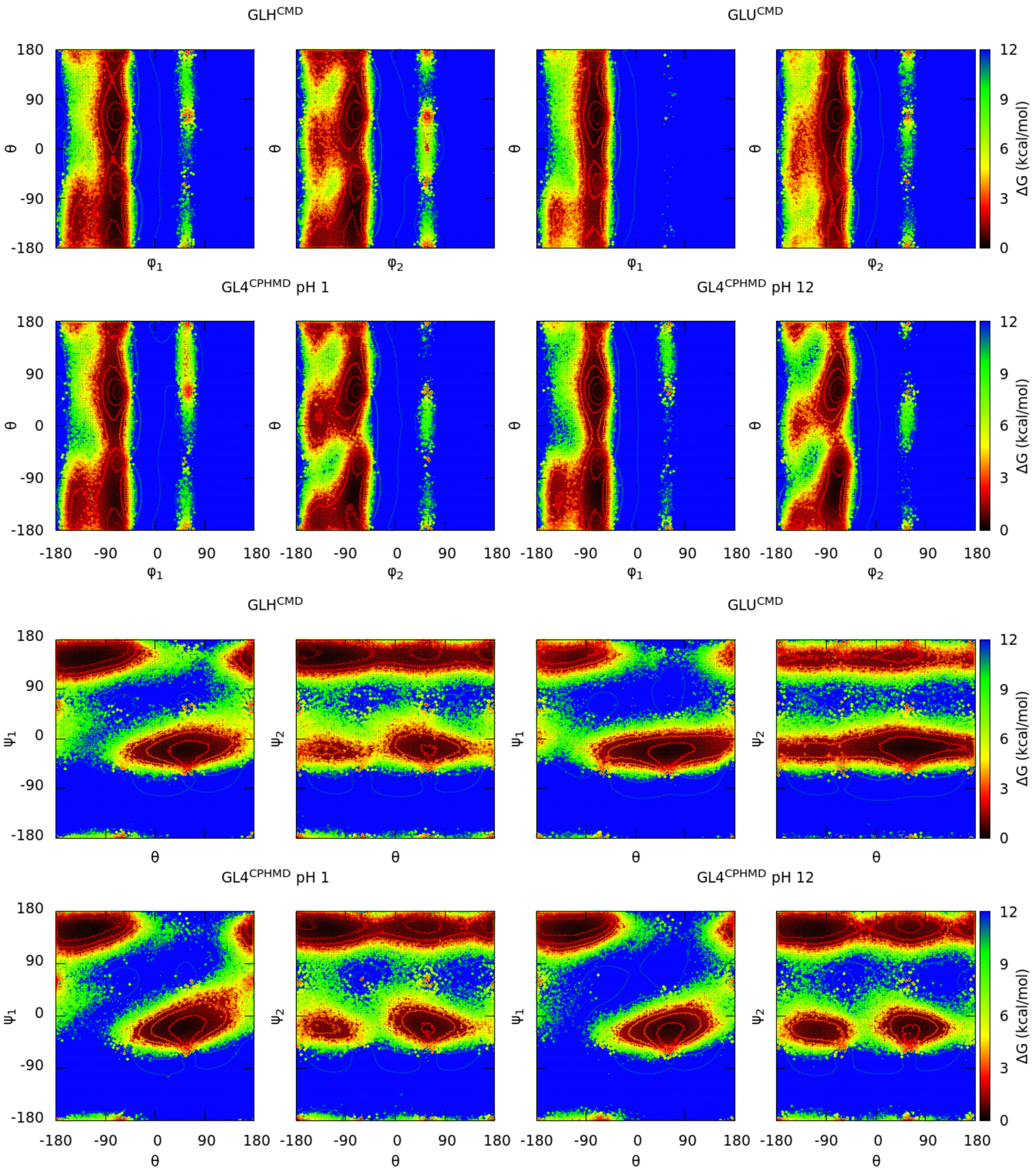

Figure S18. Gibbs free energies in the sidechain-orientation space of the blocked Glu_2 tripeptide. The labelling indicates the residue, the simulation method (in the superscript) and the pH (only for the CPHMD simulations). Four sets of dihedral angles are used in this plot, using the θ dihedral angle (CE1-CA-CA-CE1) in conjunction with the phi (φ) or psi (ψ) of each monomer (φ_1/ψ_1 from N-terminal amino acid; φ_2/ψ_2 from the C-terminal amino acid). Protonated forms are in the left and deprotonated ones in the right. Solid lines indicate an increase of 0.6 kcal/mol of the energy values.

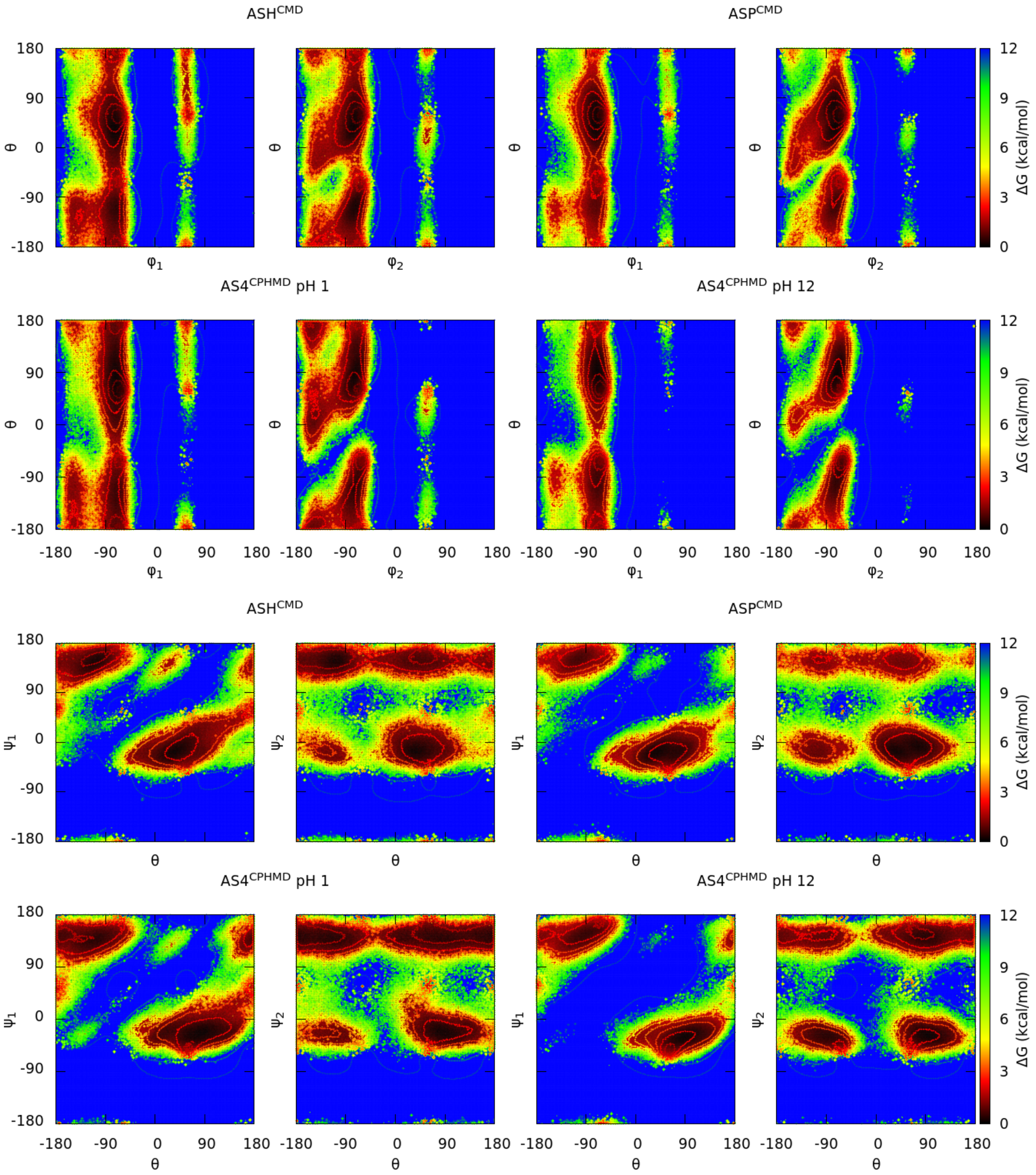

Figure S19. Gibbs free energies in the sidechain-orientation space of the blocked Asp_2 tripeptide. The labelling indicates the residue, the simulation method (in the superscript) and the pH (only for the CPHMD simulations). Four sets of dihedral angles are used in this plot, using the θ dihedral angle (CE1-CA-CA-CE1) in conjunction with the phi (φ) or psi (ψ) of each monomer (φ_1/ψ_1 from N-terminal amino acid; φ_2/ψ_2 from the C-terminal amino acid). Protonated forms are in the left and deprotonated ones in the right. Solid lines indicate an increase of 0.6 kcal/mol of the energy values.

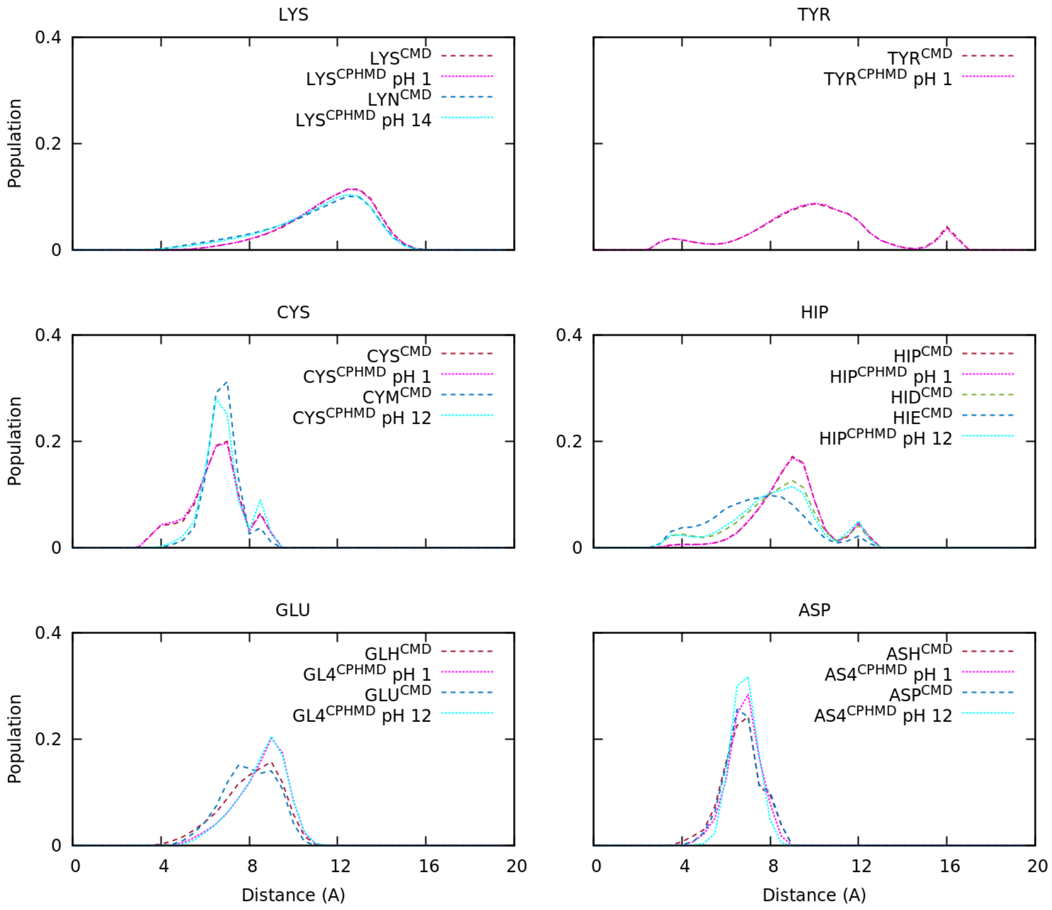

Figure S20. Distribution of the atomic distance between the atoms of the side chain selected for the construction of the θ dihedral angle.

ATOM	GLH	GLU	pH12	P-sO_2	P-aO_2	P-sO_1	P-aO_1	ATOM	ASH	ASP	pH12	P-sO_2	P-aO_2	P-sO_1	cP-aO_1
**N**	-0,4157	-0,5163	-0,4157	-0,4157	-0,4157	-0,4157	-0,4157	**N**	-0,4157	-0,5163	-0,4157	-0,4157	-0,4157	-0,4157	-0,4157
**H**	0,2719	-0,2936	0,2719	0,2719	0,2719	0,2719	0,2719	**H**	0,2719	0,2936	0,2719	0,2719	0,2719	0,2719	0,2719
**CA**	0,0145	-0,0397	0,0145	0,0145	0,0145	0,0145	0,0145	**CA**	0,0341	-0,0381	0,0341	0,0341	0,0341	0,0341	0,0341
**HA**	0,0779	0,1105	0,0779	0,0779	0,0779	0,0779	0,0779	**HA**	0,0864	-0,088	0,0864	0,0864	0,0864	0,0864	0,0864
**CB**	-0,0071	0,056	-0,0398	-0,0071	-0,0071	-0,0071	-0,0071	**CB**	-0,0316	-0,0303	-0,1783	-0,0316	-0,0316	-0,0316	-0,0316
**HB2**	0,0256	-0,0173	-0,0173	0,0256	0,0256	0,0256	0,0256	**HB2**	0,0488	-0,0122	-0,0122	0,0488	0,0488	0,0488	0,0488
**HB3**	0,0256	-0,0173	-0,0173	0,0256	0,0256	0,0256	0,0256	**HB3**	0,0488	-0,0122	-0,0122	0,0488	0,0488	0,0488	0,0488
**CG**	-0,0174	0,0136	0,0136	-0,0174	-0,0174	-0,0174	-0,0174	**CG**	0,6462	0,7994	0,7994	0,6462	0,6462	0,6462	0,6462
**HG2**	0,0430	-0,0425	-0,0425	0,0430	0,0430	0,0430	0,0430	**OD1**	-0,5554	-0,8014	-0,8014	-0,5554	-0,5554	-0,5554	-0,5554
**HG3**	0,0430	-0,0425	-0,0425	0,0430	0,0430	0,0430	0,0430	**OD2**	-0,6376	-0,8014	-0,8014	-0,6376	-0,6376	-0,6376	-0,6376
**CD**	0,6801	0,8054	0,8054	0,6801	0,6801	0,6801	0,6801	**HD21**	0,4747	-	0,0000	0,4747	0,0000	0,0000	0,0000
**OE1**	-0,5838	-0,8188	-0,8188	-0,5838	-0,5838	-0,5838	-0,5838	**C**	0,5973	0,5366	0,5973	0,5973	0,5973	0,5973	0,5973
**OE2**	-0,6511	-0,8188	-0,8188	-0,6511	-0,6511	-0,6511	-0,6511	**O**	-0,5679	-0,5819	-0,5679	-0,5679	-0,5679	-0,5679	-0,5679
**HE2**	0,4641	-	0,0000	0,4641	0,0000	0,0000	0,0000	**HD22**	-	-	0,0000	0,0000	0,4747	0,0000	0,0000
**C**	0,5973	0,5366	0,5973	0,5973	0,5973	0,5973	0,5973	**HD11**	-	-	0,0000	0,0000	0,0000	0,4747	0,0000
**O**	-0,5679	-0,5819	-0,5679	-0,5679	-0,5679	-0,5679	-0,5679	**HD12**	-	-	0,0000	0,0000	0,0000	0,0000	0,4747
**HE22**	-	-	0,0000	0,0000	0,4641	0,0000	0,0000								
**HE11**	-	-	0,0000	0,0000	0,0000	0,4641	0,0000								
**HE12**	-	-	0,0000	0,0000	0,0000	0,0000	0,4641								

Table S1. Partial charges of the protonated and deprotonated forms of the Glu and Asp amino acids in CMD and CPHMD simulations. pH(X) and p-(X) refers to the partial charges used in the CPHMD method while other labels correspond to the CMD residues. Both Glu and Asp amino acids have four protonated states: the syn- (P-sO_x) and anti- (P-aO_X) position on the two oxygens (O_1 or O_2) of the carboxyl group.

ATOM	LYN	LYS	pH1	pH14
**N**	-0,416	-0,3479	-0,3479	-0,3479
**H**	0,272	0,2747	0,2747	0,2747
**CA**	-0,072	-0,2400	-0,2400	-0,2400
**HA**	0,099	0,1426	0,1426	0,1426
**CB**	0,048	-0,0094	-0,0094	-0,1096
**HB2**	0,034	0,0362	0,0362	0,0340
**HB3**	0,034	0,0362	0,0362	0,0340
**CG**	0,06612	0,0187	0,0187	0,0661
**HG2**	0,01041	0,0103	0,0103	0,0104
**HG3**	0,01041	0,0103	0,0103	0,0104
**CD2**	-	-0,0479	-0,0479	-0,0377
**HD2**	0,01155	0,0621	0,0621	0,0115
**HD3**	0,01155	0,0621	0,0621	0,0115
**CE2**	0,32604	-0,0143	-0,0143	0,3260
**HE2**	-	0,1135	0,1135	-0,0336
**HE3**	-	0,1135	0,1135	-0,0336
**NZ**	-	-0,3854	-0,3854	-1,0358
**HZ1**	-	0,3400	0,3400	0,0000
**HZ2**	0,38604	0,3400	0,3400	0,3860
**HZ3**	0,38604	0,3400	0,3400	0,3860
**C**	0,5973	0,7341	0,7341	0,7341
**O**	-0,5679	-0,5894	-0,5894	-0,5894

ATOM	TYR	pH1
N	-0,4157	-0,4157
H	0,2719	0,2719
CA	-0,0014	-0,0014
HA	0,0876	0,0876
CB	-0,0152	-0,0152
HB2	0,0295	0,0295
HB3	0,0295	0,0295
CG	-0,0011	-0,0011
CD1	-0,1906	-0,1906
HD1	0,1699	0,1699
CE1	-0,2341	-0,2341
HE1	0,1656	0,1656
CZ	0,3226	0,3226
OH	-0,5579	-0,5579
HH	0,3992	0,3992
CE2	-0,2341	-0,2341
HE2	0,1656	0,1656
CD2	-0,1906	-0,1906
HD2	0,1699	0,1699
C	0,5973	0,5973
OH	-0,5679	-0,5679

ATOM	CYM	CYS	pH1	pH12
N	-0,4160	-0,4160	-0,4160	-0,4160
H	0,2720	0,2720	0,2720	0,2720
CA	-0,0350	0,0210	0,0210	0,0210
HA	0,0510	0,1120	0,1120	0,1120
CB	-0,2410	-0,1230	-0,1230	-0,3590
HB2	0,1120	0,1110	0,1110	0,1110
HB3	0,1122	0,1112	0,1112	0,1112
SG	-0,8844	-0,3119	-0,3119	-0,8844
HG	-	0,1933	0,1933	0,0000
C	0,5973	0,5973	0,5973	0,5973
O	-0,5679	-0,5679	-0,5679	-0,5679

ATOM	HID	HIE	HIP	pH1	pH12ε	pH12δ
**N**	-0,416	-0,4160	-0,3480	-0,3480	-0,3480	*-0,348*
**H**	0,2720	0,2720	0,2750	0,2750	0,2750	*0,2750*
**CA**	0,0190	-0,0580	-0,1350	-0,1350	-0,1350	*-0,1350*
**HA**	0,0880	0,1360	0,1210	0,1210	0,1210	*0,1210*
**CB**	-0,0460	-0,0070	-0,0410	-0,0410	-0,1110	*-0,1012*
**HB2**	0,0400	0,0370	0,0810	0,0810	0,0402	*0,0367*
**HB3**	0,0402	0,0367	0,0810	0,0810	0,0402	*0,0367*
**CG**	-0,0266	0,1868	-0,0012	-0,0012	-0,0266	*0,1868*
**ND1**	-0,3811	-0,5432	-0,1513	-0,1513	-0,3811	*-0,5432*
**HD1**	0,3649	-	0,3866	0,3866	0,3649	*0,0000*
**CE1**	0,2057	0,1635	-0,0170	-	0,2057	*0,1635*
**HE1**	0,1392	0,1435	0,2681	0,2681	0,1390	*0,1435*
**NE2**	-0,5727	-0,2795	-0,1718	-0,1718	-0,5727	*-0,2795*
**HE2**	-	0,3339	0,3911	0,3911	0,0000	*0,3339*
**CD2**	0,1292	-0,2207	-0,1141	-0,1141	0,1292	*-0,2207*
**HD2**	0,1147	0,1862	0,2317	0,2317	0,1147	*0,8620*
**C**	0,5973	0,5973	0,7341	0,7341	0,7341	*0,7341*
**O**	-0,5679	-0,5679	-0,5894	-0,5894	-0,5894	*-0,5894*

Table S2. Partial charges of the protonated and deprotonated forms of the Glu and Asp amino acids in CMD and CPHMD simulations. pH(X) and p-(X) refers to the partial charges used in the

CPHMD method while other labels correspond to the CMD residues. The histidine amino acid has two states in the neutral form: the ε- (pH12- ε) and δ- (pH12-δ) state.

System	Atomic distance		Dihedral angle	
**LYS**	LYS^{CMD}	11.89 ± 1.90	NZ-NZ	NZ-CA-CA-NZ
	LYS$^{CPHMD_{1}}$	11.87 ± 1.90		
	LYN^{CMD}	11.30 ± 2.34		
	LYS$^{CPHMD_{14}}$	11.39 ± 2.22		
**TYR**	TYR^{CMD}	10.13 ± 3.02	OH-OH	OH-CA-CA-OH
	TYR$^{CPHMD_{1}}$	10.07 ± 2.96		
**CYS**	CYS^{CMD}	6.71 ± 1.24	SG-SG	SG-CA-CA-SG
	CYS$^{CPHMD_{1}}$	6.68 ± 1.23		
	CYM^{CMD}	7.04 ± 0.71		
	CYS$^{CPHMD_{12}}$	7.08 ± 0.91		
**HIP**	HIP^{CMD}	9.10 ± 1.60	CE1-CE1	CE1-CA-CA-CE1
	HIP$^{CPHMD_{1}}$	9.12 ± 1.58		
	HIE^{CMD}	7.63 ± 2.02		
	HID^{CMD}	8.49 ± 2.04		
	HIP$^{CPHMD_{12}}$	8.42 ± 2.07		
**GLU**	GLH^{CMD}	8.34 ± 1.31	CD-CD	CD-CA-CA-CD
	GL4$^{CPHMD_{1}}$	8.75 ± 1.15		
	GLU^{CMD}	8.23 ± 1.17		
	GL4$^{CPHMD_{12}}$	8.79 ± 1.10		
**ASP**	ASH^{CMD}	6.95 ± 0.89	CG-CG	CG-CA-CACG
	AS4$^{CPHMD_{1}}$	7.05 ± 0.74		
	ASP^{CMD}	7.02 ± 0.81		
	AS4$^{CPHMD_{12}}$	7.06 ± 0.57		

Table S3. Averages and standard deviations of the interatomic distance using the selected atoms at the extreme of the side chains. The construction of the θ angle for each amino acid is also indicated.

REFERENCES

1. Rubio-Martinez, J.; Tomas, M.S.; Perez, J.J. Effect of the solvent on the conformational behavior of the alanine dipeptide deduced from MD simulations. *J. Mol. Graph. Model.* **2017**, *78*, 118–128, doi:10.1016/j.jmgm.2017.10.005.
